# Pesticide distribution and depletion kinetic determination in honey and beeswax: Model for pesticide occurrence and distribution in beehive products

**DOI:** 10.1371/journal.pone.0212631

**Published:** 2019-02-20

**Authors:** Jakob A. Shimshoni, Roy Sperling, Muhammad Massarwa, Yaira Chen, Vijayakumar Bommuraj, Mikhail Borisover, Shimon Barel

**Affiliations:** 1 Department of Food Quality & Safety, Institute for Postharvest and Food Sciences, Agricultural Research Organization, Volcani Center, Rishon Letzion, Israel; 2 Department of Instrumental Analytic, Bilacon GMbH, Berlin, Germany; 3 Institute of Plant Protection and Inspection Services, Department of Feed Quality Laboratory, Ministry of Agriculture, Rishon Letzion, Israel; 4 Department of Soil Chemistry, Plant Nutrition and Microbiology, Institute of Soil, Water and Environmental Sciences, Agricultural Research Organization, Volcani Center, Rishon Letzion, Israel; 5 Department of Toxicology, Kimron Veterinary Institute, Bet Dagan, Israel; University of California San Diego, UNITED STATES

## Abstract

Beehive products such as honey, beeswax and recently pollen have been regarded for many years as appropriate sentinels for environmental pesticide pollutions. However, despite yearly application of hundreds of approved pesticides in agricultural fields, only a minor fraction of these organic compounds were actually detected in honey and beeswax samples. This observation has led us to question the general suitability of beehive products as a sentinel for synthetic organic pesticides applied in the field. The aim of the present study was to experimentally determine the distribution (logarithmic ratio of beeswax to honey pesticide concentration, LogD) and depletion kinetics (half-life) of selected pesticides in honey and beeswax as a measure of the latter matrixes to serve as a pesticide sentinel. The obtained parameters were used to extrapolate to pesticide burden in honey and beeswax samples collected from German and Israeli apiaries. In addition, we aimed to establish a mathematical model, enabling us to predict distribution of selected pesticides between honey to beeswax, by utilizing simple substance descriptors, namely, octanol/water partitioning coefficient, molar weight and Henry coefficient. Based on the present results, it appears that pesticides with LogD values > 1 and half-life in beeswax > 1 day, were likely to accumulate and detected in beeswax samples, and less likely to be found in honey. On the other hand, pesticides with negative LogD values were highly likely to be found in honey and less so in beeswax samples. Finally, pesticides with LogD values between 0–1 were expected to be found in both matrixes. The developed model was successfully applied to predict LogD values, thereby identifying octanol/water partitioning and molar weight as the most prominent substance descriptors, which affect pesticide distribution between honey and beeswax.

## Introduction

Honeybees are crucial for the pollination of agricultural crops and wild plants, helping to ensure food security and maintain biodiversity [1]. Yet numerous studies clearly indicated that honeybee populations are increasingly threatened around the world, mostly due to exposure to various pesticides such neonicotinoids and pyrethroids as well as due to biotic stressors such as fungal, bacterial, viral and parasitic infestation [2–4]. Pesticide residues are frequently encountered in beehives as a result of pesticide carryover by honeybees, foraging on pesticide treated agricultural fields, public gardens or due to direct introduction of acaricides into beehives [4]. Pesticides are also easily spread throughout the beehive and can distribute between comb beeswax, beebread and honey, which may lead to exposure of developing brood and honey stores, potentially resulting in increased brood mortality [4–6].

Pesticides tendency to partition and accumulate in beehive products such as beeswax and honey is associated with the compound’s ability to interact with the beehive matrixes, which is a function of the pesticide’s physico-chemical properties. These physico-chemical properties determined by the pesticide chemical structure may often include molecular volume, water solubility, vapor pressure and lipophilicity [7–9]. Consequently, it is reasonable to assume that, the distribution of pesticides between honey and beeswax may resemble the partitioning of organic compounds in diverse biological and environmental matrices, such as complex lipids, tissues, food matter, plant and soil organic components [7–9]. These distributions were mathematically treated in the forms of multi-linear regression models relating (log) distribution coefficients to compound’s physico-chemical properties [7–9]. The latter provided insight into the underlying mechanism of interaction of the compound with the matrix in question [7–9].

Survey studies have clearly indicated that beeswax constitutes a major sink for lipophilic pesticides, while honey was shown to be less contaminated, containing mostly hydrophilic pesticides [6, 10–13]. Indeed numerous studies clearly revealed a major contamination of beeswax with various persistent lipophilic pesticides, thus suggesting beeswax as a sentinel of pesticide exposure [10–13].

Hence, honeybee products such as honey, beeswax and pollen, have been suggested as valuable sentinels for biomonitoring environmental pollutants, such as heavy metals, pesticides and other persistent organic pollutants [6, 10–17]. Multiple studies reporting the residue concentrations of pesticides in beehive products, mainly beeswax, honey and pollen, have been published in the last decade [6, 10–20]. The majority of the studies, which surveyed beeswax and honey simultaneously from the same beehives, reported lower pesticide contamination of honey as compared with that of beeswax, in which pesticide concentrations occasionally reaching values of up to several dozens of mg/kg [18–20]. Despite the yearly application of hundreds of approved pesticides in agricultural fields in Europe (e.g., 331 synthetic organic pesticides in 2016), only a small fraction of these active ingredients were actually screened in honey and beeswax samples (≤ 30%) in Europe [18–21]. Furthermore, among the screened pesticides, less than 35% have been detected in both matrixes [18–20]. This observation has led us to question the general suitability of beehive products as sentinels for synthetic organic pesticides applied in the field. A biological matrix may serve as suitable sentinel for environmental pollutants only when the pollutants in question are sampled at reasonable time intervals (namely, sampling frequency < 4 * pesticide half-life), characterized by a low vapor pressure, possessing a favorable distribution tendency towards the non-gaseous matrix in question and exhibiting a relative chemical/metabolic stability (expressed as half-life) [22, 23]. Moreover, a sensitive and robust analytical method is prerequisite for the detection of the pollutant in question at environmentally relevant concentrations. Hence, the suitability of honey and beeswax to function as sentinel for pesticides with low vapor pressure depends mostly upon the pesticide’s persistence (expressed as half-life) and the pesticides honey-beeswax distribution tendency. Consequently, the aims of the present study were to experimentally determine the distribution, expressed as the logarithmic ratio of pesticide concentration in beeswax to honey (LogD), and the depletion kinetics (half-life) of selected pesticides in beeswax and honey phases as a function of time. To the best of our knowledge, no published studies are available regarding the depletion kinetics and distribution behavior of pesticides in honey and melted beeswax, with the exception of one research study, in which the distribution of polychlorobiphenyls (PCBs), chlorobenzenes, dichlorodiphenyltrichloroethane (DDT) and its metabolites between beeswax and honey was examined [24]. Based on the data obtained, we additionally aimed to establish a multi-linear regression model, in order to predict the distribution of selected pesticides between honey and beeswax.

## Materials and methods

### 2.1 Reagents

Acetonitrile, glacial acetic acid, methanol, ethyl acetate and formic acid (all of HPLC grade) were obtained from Sigma-Aldrich (Saint Louis, MO, US). Polymerically bonded ethylenediamine-N-propyl phase (PSA) was purchased from Varian (Palo Alto, CA, USA), while anhydrous magnesium sulfate, florisil, anhydrous ammonium acetate and sodium acetate were purchased from Sigma-Aldrich (Saint Louis, MO, USA). Aqueous solutions were prepared with ultra-pure-water (Milli-Q Plus system; Millipore Corp., Billerica, MA, USA). All pesticide standards were of high purity grade (>99.0%), purchased from Sigma-Aldrich (Saint Louis, MO, USA). Individual stock solutions were prepared at 1000 mg/L in acetonitrile or methanol and stored at -20°C. The working solutions were prepared by carrying out appropriate dilutions of the stock solutions.

### 2.2 Pesticide-free honey and beeswax samples for depletion and distribution study

For the preparation of pesticide-free beeswax, 100 gr of commercially available beeswax foundation (Nir-Galim, Israel) was transferred into a 500 mL Erlenmeyer flask and inserted into a 75°C water bath. Immediately upon melting, 200 mL of acetonitrile was added to the melted beeswax and vigorously shaken for 5 min, followed by beeswax freeze-out in the fridge for 2 min. The acetonitrile supernatant was discarded and the extraction process repeated twice. Commercially available organic pesticide-free honey samples were obtained from the local supermarket (Tel Aviv, Israel). The honey and beeswax samples were stored in the dark at room temperature (25°C) until analysis.

### 2.3 Israeli honey samples collected for pesticide survey

Honeycombs (20 gr) were collected between January 2018 to July 2018 from 22 apiaries in 22 locations in Israel, thereby covering the entire Israeli landscape. The study was carried out on private land and the owner of the land gave permission to conduct the study on this site. At each apiary, three healthy bee colonies were selected for honeycomb sampling. The three honeycomb portions from each apiary were pooled together, to obtain a single representative sample. Pure honey samples were obtained by allowing honey to completely drain into a glass vessel. The samples were transported to the laboratory and stored at -20°C until analysis. The Israeli honey samples were screened for all of the listed pesticides depicted in S1 and S2 Tables.

### 2.4 Database of pesticide occurrence in beeswax and honey samples from Bilacon GMbH, Germany

Data of pesticide concentrations from German beeswax (n = 513) and honey (n = 338) samples, collected and analyzed from German apiaries from January 2015 to May 2018 and from January-May 2018, respectively, were kindly obtained from Bilacon GMbH, Berlin, Germany. The German samples were not uniformly screened for all the pesticides listed in S1 and S2 Tables; instead, only by the customer requested pesticides were analyzed for each sample.

### 2.5 Sample preparation and extraction of beeswax and honey samples

Beeswax samples were first melted in an Erlenmeyer flask (500 mL) at 75° C and hand-shaken for 2 min. Subsequently, 1 g of melted beeswax was placed into a 50 mL-conical tube. Then, 10 mL of double distilled water, 10 mL acetonitrile and internal standards were added to the conical tube. The sample mixtures were placed in a water bath at 75°C and shaken for 10 min (Thermo Scientific Shaking Water Bath TSSWB15, USA). Subsequently, the mixtures were subjected to centrifugation (Thermo Scientific Heraeus Megafuge 16, USA) for 2 min at 4700 rpm and the supernatant transferred into a new 50 mL conical tube. The removal of residual beeswax was achieved by storing the supernatant overnight at -20°C, followed by centrifugation at 13,000 rpm for 5 min to separate the precipitated beeswax from the acetonitrile supernatant. Finally, 7 ml of the supernatant was pipetted into a 15 mL-dispersive solid phase extraction tube (d-SPE tube) packed with primary secondary amines (PSA) and octadecyl (C18). The content was mix vortexed for 1 min and centrifuged at 4700 rpm for 5 min to obtain 1 mL of purified sample extract. The extracted samples were stored in glass screw capped bottles for liquid chromatography tandem mass spectrometry (LC-MS/MS) and gas chromatography tandem mass spectrometry (GC-MS/MS) analyses. The Multiple Reaction Monitoring (MRM) transition and optimized parameters for the pesticides analyzed by LC-MS/MS as well as GC-MS/MS are given in the supplemental section (S1 and S2 Tables).

Two grams of honey, 25 μL of 10 mg/L internal standard solution (S1 and S2 Tables), 10 mL of water, and 10 mL of acetonitrile were mixed in a 50 mL centrifuge tube, which were then vigorously shaken by hand until a homogeneous solution was obtained. A mixture of 4 g of anhydrous magnesium sulfate, 1 g of sodium chloride, 1 g of trisodium citrate dihydrate, and 0.5 g of disodium hydrogen citrate sesquihydrate was added to the tube. The tube was shaken vigorously by hand for 1 min and centrifuged for 5 min at 3000 rpm and 10°C. An aliquot of 6 mL of the acetonitrile phase was transferred into a 15 mL conical tube containing 900 mg of anhydrous magnesium sulfate and 150 mg of PSA. The tube was vigorously shaken by hand for 30 sec and centrifuged for 5 min at 3000 rpm at 10°C. Two milliliters of the supernatant was evaporated to dryness using a stream of nitrogen at 30°C. Subsequently, the residue was re-dissolved in 1mL of methanol/water 20:80 (v/v) and subjected to LC-MS/MS analysis.

### 2.6 Liquid chromatography tandem-mass spectrometry (LC-MS/MS) analysis

All analyses were performed on an ACQUITY UPLC (ACQUITY UPLC, XEVO TQD mass spectrometer; Waters Corp., Milford, MA, USA), equipped with a quaternary pump and membrane degasser. The separation column, Zorbax SB-C18 (2.1 × 150 mm i.d. and 3.5 μm; Agilent Technologies, Santa Clara, CA, USA), was kept at 40°C. An automatic injector was set to inject 10 μL per sample. The mobile phase components were (A) a 10 mM ammonium acetate solution in water and (B) acetonitrile with 0.1% formic acid. The gradient used, was initially set at a flow rate of 0.4 mL/min of 95% mobile phase A for 0.25 min. From 0.25 min to 7 min, a linear gradient was used up to 95% mobile phase B, which was maintained for 1 min. Then, a linear gradient was used to reach 95% mobile phase A, maintained for 1 min. Sample analyses were performed using a triple quadrupole system with positive and negative ESI. More information on the technical settings used is provided in S1 Table in the supplemental section. The analytes were monitored and quantified using MRM mode. Optimization of the MS/MS conditions, identification of the parent and product ions, as well as the selection of the cone and collision voltages, were performed with direct infusion of their individual standard solutions (S1 Table). Every individual standard pesticide solution was prepared in a concentration of 1 mg/mL in water/acetonitrile (1:1). The Masslynx software was used for the LC-MS/MS system control and data analysis.

### 2.7 Gas chromatography with tandem mass spectrometer (GC-MS/MS) analyses

The analyses were carried out on a 7890 GC equipped with a 7693B auto sampler and a 7000 series GC-MS/MS system (Agilent Technologies, Palo Alto, CA, USA). A column HP-5MS UI 15 m×0.25 mm×0.25 μm (Agilent Ultra GC column) was used to provide analyte separation. Sample injections were performed in a 7890A GC multimode inlet operated using the splitless-injection mode through an inlet liner filled with a glass wool frit (Ultra Iner liner from Agilent). The injector operating conditions were as follows: the injection volume was 2 μL, the injector temperature was held at 80°C during the solvent evaporation stage, then ramped up to 300°C at 600 C min^−1^ and, finally, this temperature was held for 20 min. Helium, with a purity of 99.999%, was used as both the carrier gas and the quenching gas, and nitrogen with a purity of 99.999% as the collision gas. The oven temperature program was set as follows: 70°C for 1 min, programmed to 150°C at 50°C min^−1^, then to 200°C at 6°C min^−1^ and, finally, 280°C at 16°C min^−1^ (4.07 min). The total run time was 20 min plus three additional min to backflush at 280°C. The triple quadrupole mass spectrometer was operated using electron impact ionization (EI) and in the Selected reaction monitoring (SRM) mode. The temperatures of the transfer line, ion source and quadrupole 1 and 2 were 280°C, 280°C and 150°C, respectively. The analysis was performed with a solvent delay of 2 min in order to prevent instrument damage. The electron multiplier voltage was set at 1592 V. Mass peak widths were set to wide in the first and third quadrupoles. For control and data analysis, MassHunter B.05.00 software (Agilent) was used. The two most intense mass transitions and their optimal collision energies were selected for pesticide quantification (S2 Table). The most intense product was selected as the quantifier ion and the second as the qualifier ion. The collision gas flow was 1.5 mL min^−1^ and the quenching gas flow was 2.25 mL min^−1^. A 4-time-segment SRM method was created to obtain adequate sensitivity and signal-to-noise (S/N) relationship; the cycle time for each segment was set between 200 and 250 msec.

### 2.8 Pesticide depletion kinetic study in beeswax and honey

The stability of 27 pesticides commonly applied in Israel was studied in commercial pesticide-free beeswax (purified) and local honey. The following pesticides were included in the present study: acetamiprid, alachlor, amitraz, atrazine, bifenthrin, boscalid, bromopropylate, carbendazim, chlorantraniliprole, chlorpyrifos, clothianidin, coumaphos, cypermethrin, diuron, fenbutatin oxide, imidacloprid, iprodione, malathion, metconazole, metolachlor, oxadiazon, oxyfluorfen, pyraclostrobin, tau- fluvalinate, tebuconazole, thiacloprid, thiamethoxam (Table 1). Each pesticide was spiked separately, by adding 50 μL of pesticide working solution (100 mg/L or 10 mg/L) in acetonitrile at 75°C, into 10 gr honey or 10 gr beeswax, yielding final concentrations of 0.5 mg/kg or 0.05 mg/kg, respectively. For each pesticide, the depletion kinetic was repeated separately three times for three consecutive weeks. The depletion kinetics was studied at 75°C and the pesticide concentration was monitored over a period of 7 days. The concentrations tested, were within the concentration range found for all the pesticides analyzed herein in beeswax and/or in honey, which concomitantly enabled the quantification of residual concentrations over a 7-day period [10–20]. The tenfold lower concentration of 0.05 mg/kg in honey and beeswax was too low to enable its detection after 1–8 hr incubation at 75°C, thus precluding its usage for evaluating its terminal half-life. The decline in concentrations was determined by LC-MS/MS or GC-MS/MS at the following time points: 5 min, 1 hr, 8 hr, 24 hr and 168 hr (S1 and S2 Tables) [25]. The initial honey pH, before study onset, was 3.75 ± 0.2 and declined to 3.41 ± 0.15 after 7 days of incubation at 75°C. The honey pH was measured at room temperature, diluted 1: 1 with double distilled water.

**Table 1:** Depletion t_1/2_ and LogD values of selected pesticides in honey and beeswax.

Pesticide*	Honey		Beeswax		LogD^a^	Unpaired two tailed T-Test
	k^b^ (day^-1^) ± SD**	t_1/2_ (day)	k (day^-1^) ± SD	t_1/2_ (day)		p-value (t value)
Acetamiprid	0.50 ± 0.04	1.4	0.19 ± 0.03	3.6	-0.75	0.0004 (10.74)
Alachlor	1.16 ± 0.17	0.6	0.12 ± 0.01	6	2.36	0.0005 (10.57)
DMF***	0.41 ± 0.05	1.7	0.12 ± 0.03	5.9	-0.67	0.001 (8.61)
DMPF***	1.39 ± 0.4	0.5	3.47 ± 0.5	0.2	0.54	0.0049 (5.62)
Atrazine	1.73 ± 0.6	0.4	0.58 ± 0.04	1.2	2.08	0.029 (3.31)
Bifenthrin	0.15 ± 0.04	4.7	0.05 ± 0.007	15.2	ND^c^	0.013 (4.26)
Boscalid	0.41 ± 0.05	1.7	0.04 ± 0.006	16	1.89	0.0002 (12.72)
Bromopropylate	0.46 ± 0.07	1.5	0.27 ± 0.03	2.6	ND	0.012 (4.32)
Carbendazim	0.99 ± 0.2	0.7	0.53 ± 0.08	1.3	0.78	0.021 (3.69)
Chlorantraniliprole	0.87 ± 0.1	0.8	0.12 ± 0.05	5.9	1.32	0.0003 (11.62)
Chlorpyrifos	0.36 ± 0.07	1.9	0.08 ± 0.01	8.8	2.75	0.0024 (6.85)
Clothianidin	0.20 ± 0.03	3.6	0.10 ± 0.02	6.9	-1.15	0.0086 (4.80)
Coumaphos	0.46 ± 0.05	1.5	0.08 ± 0.009	8.3	2.34	0.0002 (12.95)
Cypermethrin	0.14 ± 0.02	4.9	0.01 ± 0.003	96.3	ND	0.004 (11.13)
Diuron	1.16 ± 0.40	0.6	10^4^ ± 598^d^	0.025	ND	<0.0001 (> 28.96)
Fenbutatin oxide	0.13 ± 0.03	5.4	0.02 ± 0.005	32.1	1.21	0.0033 (6.26)
Imidacloprid	0.24 ± 0.03	2.9	0.17 ± 0.01	4.1	-1.04	0.018 (3.83)
Iprodione	0.77 ± 0.09	0.9	0.14 ± 0.04	5.0	ND	0.0004 (11.07)
Metolachlor	1.16 ± 0.30	0.6	0.14 ± 0.04	5.0	2.28	0.0043 (5.83)
Metconazole	0.41 ± 0.07	1.7	0.11 ± 0.03	6.4	2.11	0.0024 (6.83)
Oxadiazon	0.22 ± 0.01	3.2	0.12 ± 0.02	5.9	ND	0.0015 (7.74)
Oxyfluorfen	0.50 ± 0.06	1.4	0.28 ± 0.04	2.5	ND	0.0062 (5.28)
tau-Fluvalinate	0.27 ± 0.04	2.6	0.01 ± 0.002	48.1	ND	0.0004 (11.2)
Tebuconazole	0.26 ± 0.05	2.7	0.08 ± 0.01	9	ND	0.0036 (6.11)
Thiacloprid	0.53 ± 0.06	1.3	0.30 ± 0.02	2.3	-0.64	0.0032 (6.29)
Thiamethoxam	0.15 ± 0.02	4.6	0.12 ± 0.03	5.8	-2.06	0.223 (1.44)

^a^LogD, Log distribution ratio, calculated as the logarithmic ratio of pesticide concentration in beeswax to honey at equilibrium.

^b^k, depletion rate constant obtained from the terminal slope of the pesticide concentration vs. time semi logarithmic plot. Mean k values were determined from three independent repeats.

^c^ND, not detectable. LogD could not be determined, due to undetectable concentrations in honey.

^d^Depletion kinetics of diuron in beeswax is of zero order, hence the half-life depends on the initial concentration (C_0_) and on the linear slope (t_1/2_ = C_0_/2*k). The k unit of diuron is μg/kg/day.

*The pesticides amitraz, malathion and pyraclostrobin were not included in Table 1, since no residual concentration in honey and beeswax were found after 5 min incubation time.

**SD, standard deviation.

***DMF and DMPF, N-(2,4-Dimethylphenyl)-formamide and N'-(2,4-Dimethylphenyl)-N-methylformamidine, respectively.

The terminal depletion rate constant (k) of the first order depletion kinetics was obtained from the least terminal slope of the semi-natural logarithmic concentration vs. time plot, utilizing WinNonline estimation program (WinNonlin version 4.1 Pharsight Co., Mountain View, CA, USA) [26]. Pesticides displaying first order depletion kinetics, the half-life time (t_1/2_) was calculated according to the equation

$$t_{1/2}=Ln\left(2\right)/k.$$ (1)

For the pesticides following zero-order depletion kinetics, the rate constant was obtained from the slope of the concentration vs. time regression line, and the half-life time was calculated according to the equation:

$$t_{1/2}=C_{0}/\left(2*k\right);$$ (2)

C_0_, concentration at time zero. The decision on the kinetic model best describing the pesticide behavior was based on the R^2^ value as well as the sum of square standard error.

### 2.9 Distribution kinetics of pesticides between melted beeswax and honey

The distribution of the selected pesticides was determined between pesticide-free materials, i.e., honey (9.5 gr) and melted beeswax (9.5 gr), at 75°C, utilizing a modified version of the Shake Flask Method [27]. Beeswax at room temperature is solid, honey is highly viscous, and therefore a rapid diffusion between both phases is only feasible at temperatures of at least 10°C higher than the beeswax melting point (65°C). The honey pH value, measured at room temperature, dropped after 7 days of incubation at 75°C, from 3.75 ± 0.2 to 3.41 ± 0.15. The depletion kinetic in honey and beeswax was determined simultaneously and separately for each pesticide, after spiking the honey phase with 50 μL of 100 mg/L pesticide stock solution in acetonitrile and allowing each pesticide to distribute between the two phases until an equilibrium was attained. For each pesticide, the distribution kinetics was repeated separately three times for three consecutive weeks. The equilibrium was characterized by a parallel decline of the pesticide in both phases, so that the concentration ratio C_beeswax_/C_honey_ remained constant over time (S3 Table). The two-phase system was established as follows: triplicates of five glass-tubes of 20 mL volume containing 9.5 gr honey were prepared and placed in a water bath shaker for 30 min at 75°C. Subsequently, the honey samples were spiked with 4.75 μL of pesticide 1 mg/mL stock solution and mixed vortexed for 10 sec, yielding a final concentration of 0.5 mg/kg. Stock solutions were freshly prepared in acetonitrile on the same day of the experiment. Then, a pre-weighted (9.5 gr) and at 75°C melted beeswax was immediately inserted into each honey-containing glass-tube and mixed vortex for 10 sec, before placing back into the water bath shaker. Both phases at 75°C nearly filled the entire volume of the test vessels, thereby reducing the gas phase volume to less than 1 mL. In order to monitor the kinetics of pesticide distribution between phases, at each of the following time points, 20 min, 1 hr, 8 hr, 24 hr and 168 hr, the glass-tubes were subjected to centrifugation at 5000 rpm at room temperature for 2 min. The two phases were separated and the wax phase washed with 100 mL doubled distilled water 3 times in order to removal residual honey before analysis. The beeswax to honey Log distribution coefficient (LogD) was experimentally determined as the Log ratio of the concentrations of the dissolved pesticide in the two immiscible phases, namely beeswax and honey.

### 2.10 Prediction of LogD values using multi-linear regression model

In order to develop a model for the prediction of LogD, an inventory of available substance descriptors closely associated with partitioning behavior between different phases and including log octanol/water partitioning coefficient (LogP), log Henry coefficient (H; calculated from water solubility and vapor pressure) and molar weight (MW) was made (Table 2) [28, 29]. Due to the lack of information regarding the pKa values of the pesticides studied herein, the latter could not be used as a substance descriptor, despite the potential impact of honey’s pH (3.7–3.4, measured as aqueous honey dilution) on the ionization state. In agreement with previous studies, a multi-linear regression model approach was utilized [7–9, 28, 29]. The following multi-linear model was introduced:

LogD=a+b*LogP+c*MW+d*LogH. (3)

**Table 2:** Substance descriptors of selected pesticides for LogD modeling.

	LogP^b^	Molar weight (g/mol)	Predicted vapor pressure^c^ at 25°C (mm Hg)	Water solubility^d^ at 25°C (mol/m^3^)	Henry’s Law Constant (H)^e^ (mm Hg *m^3^/mol)	pka^f^
Acetamiprid	0.8	222.7	44*10^−6^	1.9*10^−2^	0.002	0.7
Alachlor	3.1	269.7	22*10^−6^	8.9*10^−4^	0.025	-
DMF^a^	1.5	149.2	464*10^−6^	2.4*10^−2^	0.019	-
Atrazine	2.7	215.7	289*10^−9^	1.3*10^−4^	0.002	1.68
Boscalid	2.9	343.2	54*10^−9^	13*10^−6^	0.004	-
Carbendazim	1.5	191.2	75*10^−9^	4.2*10^−6^	0.018	4.2
Chlorantraniliprole	2.8	483.2	12*10^−13^	2.0*10^−6^	0.000001	10.8
Chlorpyrifos	4.7	350.6	202*10^−7^	3.2*10^−6^	6.31	-
Clothianidin	0.9	249.7	98*10^−9^	13*10^−4^	0.0001	11.1
Coumaphos	4.1	362.7	97*10^−9^	4.1*10^−6^	0.024	-
Fenbutatin oxide	5.0	1052.7	18*10^−10^	1.2*10^−8^	0.15	-
Imidacloprid	0.6	255.6	40*10^−07^	2.4*10^−3^	0.0017	1.5
Metconazole	3.8	319.8	92*10^−7^	4.7*10^−5^	0.20	-
Metolachlor	3.4	283.8	313*10^−7^	1.8*10^−3^	0.017	-
Thiacloprid	1.2	252.7	378*10^−9^	7.3*10^−4^	0.0005	-
Thiamethoxam	-0.1	291.7	514*10^−9^	14*10^−3^	0.00004	-

^a^ N-(2,4-Dimethylphenyl)-formamide.

^b^LogP values were experimentally determined and obtained from the Environmental Protection Agency (EPA) website: https://comptox.epa.gov/dashboard/dsstoxdb/advanced_search and PubChem website: https://www.ncbi.nlm.nih.gov/pccompound

^c^Predicted vapor pressure retrieved from EPA Chemistry Dashboard: https://comptox.epa.gov/dashboard/

^d^Water molar solubility was retrieved from EPA Chemistry Dashboard: https://comptox.epa.gov/dashboard/ and PubChem Open Chemistry Database: https://pubchem.ncbi.nlm.nih.gov/

^e^Henry’s Law Constant (H) is calculated as the ratio of a compound’s abundance in the gas phase to that in the aqueous phase at equilibrium: H = P_i_/C_w_; where P_i_ is the partial pressure of the chemical in the gas phase and C_w_ is its molar solubility in water.

^f^pKa values (determined at 25°C) were obtained from the Toxicology Data Network website: https://toxnet.nlm.nih.gov/newtoxnet/hsdb.htm

### 2.11. Risk assessment of contaminated honey to forager bees

In the present study, risk assessment was applied solely to forager honeybees, since forager bees display the highest honey consumption rate (80 mg honey and/or nectar per day) [30]. Furthermore, the mean oral LD_50_ values were only available for adult honeybees and were retrieved from the Pesticide Properties Database website, University of Hertfordshire. The following equation was utilized for calculating risk probability as a function of pesticide occurrence rate (% of positive samples within the sample set) and the daily residue dose [6]: (4) Risk [%] = pesticide occurrence rate [%] * daily residue dose [μg]/LD_50_ [μg/bee].

The daily residue dose was obtained from the product of the mean pesticide residue concentration in honey and the mean daily honey consumption of forager bees.

Risk values below 0.1% are considered negligible, while risk values between 0.1% -1% are considered of low relevance to honeybees. Risks between 1%-5% are considered moderate, while risk values above 5% are considered high for honeybee’s wellbeing [6]. For additional information, the reader may refer to the study published by Sanchez-Bayo [6].

### 2.12 Statistical analysis

Wherever applicable, numerical results were presented as means and standard deviation of the mean. Descriptive statistics were performed using statistical analysis program (GraphPad Prism version 5.00 for Windows, GraphPad Software, San-Diego, CA, USA). Regression analysis between octanol/water partition coefficient (LogP) and beeswax/honey distribution coefficient (LogD) was performed by multi-linear regression analysis, by utilizing Statistica^R^ software (StatSoft, Inc., Tulsa, OK, USA). The change of LogD values as a function of time, essential for determining time-independent LogD values, was evaluated by ANOVA linear regression analysis, utilizing the Excel software (Microsoft Excel 2016), in which the difference of the slope from zero was set as the Null hypothesis, at a significance level of p ≤ 0.05. The mean half -life values in beeswax and honey were compared for each pesticide separately by applying the unpaired two-tailed t-test at a significance level of p ≤ 0.05. The unpaired two-tailed t-test was performed using the statistical analysis program (GraphPad Prism version 5.00 for Windows, GraphPad Software, San Diego, CA, USA). The regression analysis was performed using the software Statistica^R^ (StatSoft, Inc., Tulsa, OK, USA) and characterized by square of correlation coefficient (R^2^) and the Fischer F-criterion.

## Results

### 3.1. Depletion kinetics of pesticides in beeswax and honey

**Fig 1:**
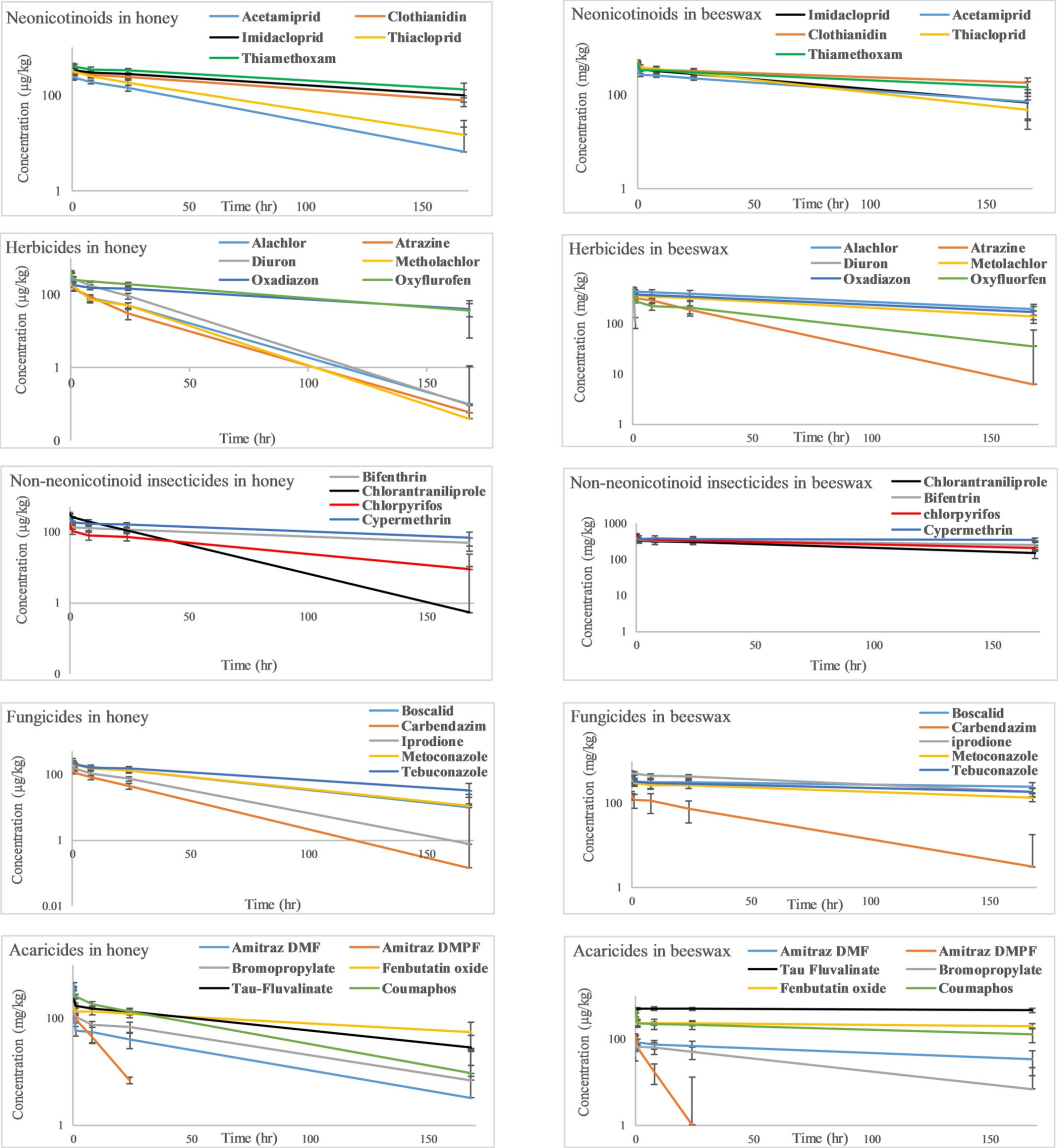
Semi-logarithmic plot of pesticides depletion kinetics in honey and beeswax, determined separately for each phase over a period of 7 days at 75°C.

Fig 1 depicts the depletion kinetics of 27 tested pesticides in beeswax and honey at 75°C, over a period of seven consecutive days.

The pesticide half-life values in beeswax were significantly longer than the half-life values in honey for most pesticides tested in the present study (p ≤ 0.05), with the exception of thiamethoxam, displaying comparable half-life values in both phases and diuron, revealing significantly longer half-life in honey than in beeswax (Table 1). The depletion kinetics of all the neonicotinoids in both phases followed first order kinetics (Fig 1, Table 1). The neonicotinoids, acetamiprid, clothianidin, imidacloprid and thiacloprid, displayed 1.4–2.5 times longer t_1/2_ in beeswax as compared to honey (Table 1). The most persistent neonicotinoids in honey and beeswax were thiamethoxam and clothianidin (Table 1).

The depletion kinetics of the herbicides, alachlor, atrazine, metolachlor, oxadiazon and oxyfluorfen followed first order kinetics in both beeswax and honey with t_1/2_ ranging between 1.2–6 days and 0.4–3.2 days, respectively (Table 1). While diuron followed first order depletion kinetics in honey, its depletion kinetics in beeswax was accurately described by a zero-order kinetics (Fig 1, Table 1). Diuron was the only herbicide, which displayed slower depletion in honey than in beeswax, with a t_1/2_ in honey being 24 times longer than in beeswax (Table 1). Oxyfluorfen, oxadiazon, metolachlor, atrazine and alachlor, reveled higher persistence in beeswax than in honey with t_1/2_ in beeswax being 1.8–10 times higher than in honey (Table 1). The fungicides boscalid, carbendazim, iprodione, metconazole and tebuconazole followed first order depletion kinetics with t_1/2_ being 1.8–10 times higher in beeswax than in honey. Boscalid displayed the longest t_1/2_ in beeswax of 16 days, followed by tebuconazole (9 days), metconazole (6.4 days), iprodione (5 days) and carbendazim (1.3 days). In honey, tebuconazole was the most persistent fungicide (t_1/2_ = 2.7 days) followed by metconazole (1.7 days), boscalid (1.6 days), iprodione (0.9 days) and carbendazim (0.7 days). Pyraclostrobin was the only fungicide displaying rapid depletion kinetics in both phases, with no detectable concentrations within 1 min. The non-neonicotinoid insecticide group, bifenthrin, chloranthraniliprole, chlorpyrifos and cypermethrin followed first order depletion kinetics with t_1/2_ in beeswax being 3.2–20 times higher than in honey (Table 1). Cypermethrin was the most persistent insecticide in beeswax and honey (t_1/2_ of 96.3 and 4.9 days, respectively). Malathion was the only insecticide with no detectable concentrations in beeswax and honey within 1 min incubation time. The persistence of the non-neonicotinoid insecticides in beeswax and honey can be classified in a descending order as follows: cypermethrin > bifenthrin > chlorpyrifos > chloranthraniliprole > malathion. The acaricides, bromopropylate, coumaphos, fenbutatin oxide and tau-fluvalinate were more persistent in beeswax than in honey, displaying 1.2–18.5 times longer t_1/2_ as compared to honey (Table 1). Amitraz was the only acaricide undergoing complete degradation within 1 min incubation time in both phases. Its two major metabolites however, N-2,4-dimethylphenyl-N'-methylformamidine (DMPF) and N-2,4-dimethylphenylformamide (DMF), were detectable in honey up to 7 days, while in beeswax only DMF was quantifiable up to 7 days, whereas DMPF was found below its detection limit (1 μg/kg) after 24 hr incubation time (Fig 1). In honey and beeswax, DMF displayed higher persistence as compared to DMPF, with t_1/2_ in honey and beeswax being 3.4 and 29 times longer than the corresponding DMPF t_1/2_, respectively (Table 1). The complete depletion of amitraz in honey within 1 min, yielded the following major metabolite fractions (fm = 100 * [amount of metabolite in μmol]/[spiked amount of amitraz in μmol]): 71% DMF (100 * [0.012 μmol]/[0.017 μmol]) and 20% DMPF (100 * [0.012 μmol]/[0.017 μmol]). In beeswax however, DMPF constituted the major degradation fraction, namely 65% (100 * [0.0073 μmol]/[0.017 μmol]), while DMF yielded the lower fraction of 22.5% ((100 * [0.0026 μmol]/[0.017 μmol]). Hence, beeswax mostly promoted the formation of DMPF, while honey favored the formation of DMF. The most persistent acaricide in beeswax was tau-fluvalinate (t_1/2_ = 48.1 days), followed by fenbutatin oxide (t_1/2_ = 32.1 days), coumaphos (t_1/2_ = 8.3 days), bromopropylate (t_1/2_ = 2.1 days). In honey on the other hand, the most persistent acaricide was fenbutatin oxide (t_1/2_ = 5.4 days), followed by tau-fluvalinate (t_1/2_ = 2.6 days), bromopropylate (t_1/2_ = 1.8 days) and coumaphos (t_1/2_ = 1.5 days).

### 3.2. Distribution kinetics of pesticides between melted beeswax and honey

**Fig 2:**
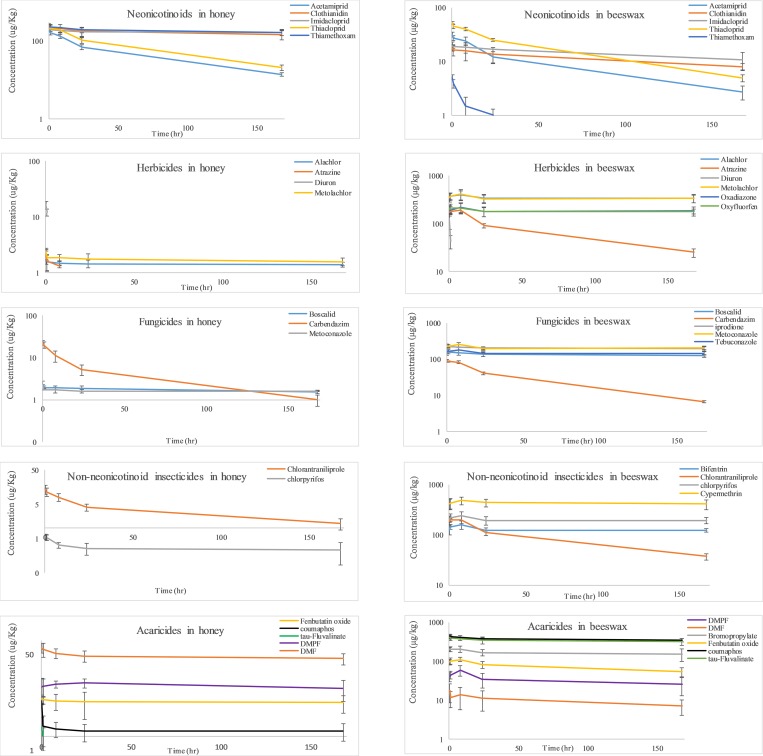
Semi-logarithmic plot of pesticides distribution between honey and beeswax over time at 75°C.

Fig 2 depicts the semi-logarithmic plots of pesticide concentration vs. time, determined in the biphasic honey-beeswax system. In honey, except for tau-fluvalinate and diuron, all pesticides tested herein, displayed on a semi-logarithmic plot a biexponential decline, which can be described by a first order biexponential equation of the form: C = A*e^-α*t^ + B*e^-β*t^, where the subscripts refer to the first and second exponential terms, respectively and A and B refer to the corresponding zero-time intercepts (Fig 2) [26]. In beeswax, on the other hand, due to initial partitioning of pesticides from honey to beeswax, most of the pesticides tested, displayed an initial increase in pesticide concentration, with maximal concentration (C_max_) attained mostly between 1–8 hours (Fig 2), followed by a biexponential decline with terminal slopes (equal to the depletion constant, k) ranging between 0.0003–0.179 hr^-1^ [26]. Diuron was the only pesticide displaying a linear decline in beeswax and honey, following zero order kinetics and best described by a linear equation of the form: C = A–B*t, where A and B denote the y-axis intercept and the linear slope, respectively (Fig 2).

In beeswax, no initial increase in neonicotinoid concentration was observed, since all of them attained C_max_, before the first sampling time point of 20 min, so that only a biexponential decline was observed, with terminal slopes ranging between 0.004–0.012 hr^-1^ (Fig 2).

The herbicide concentrations within the honey were more than 80 times lower than the corresponding concentrations in beeswax at all time points (Fig 2). Among the herbicides, diuron was the only compound fully depleted within 1 hr in honey and beeswax, following a zero-order kinetics (Fig 2). After 20 min incubation time, oxadiazon and oxyfluorfen were below their detection limit (0.1 μg/kg) in honey, while atrazine was fully depleted from honey after 8 hr incubation time (Fig 2). Alachlor and metolachlor were detectable in honey for up to 7 days, reaching their terminal slope after 1 hr (Fig 2). In beeswax on the other hands, alachlor, atrazine, metolachlor, oxadiazon and oxyfluorfen, attained C_max_ after 8hr, declining subsequently biexponentially, reaching their terminal slope after 24 hr (Fig 2).

The insecticides, bifenthrin, cypermethrin and chlorpyrifos were undetectable in honey after 20 min incubation time, while chloranthraniliprole was detectable for 7 days, declining biexponentially, achieving its terminal phase after 24 hr incubation time (Fig 2). Chloranthraniliprole concentrations in beeswax were more than 14 times higher than the corresponding honey concentrations for the duration of 7 days, reaching C_max_ at 8 hr. Cypermethrin, bifenthrin and chlorpyrifos attained C_max_ in beeswax after 8 hr, subsequently declining biexponentially, with the last terminal phase achieved after 24 hr incubation time (Fig 2).

The fungicides, tebuconazole and iprodione were below their detection limit (0.1 μg/kg) in honey after 20 min incubation time, whereas boscalid, carbendazim and metconazole were quantifiable up to 7days incubation time (Fig 2). The latter three fungicides declined in honey biexponentially, achieving their terminal slope after 8 hr (carbendazim) and 1 hr (boscalid and metconazole) (Fig 2). In beeswax, after 20 min incubation time, all fungicides concentrations were above 100 μg/L, with metconazole and tebuconazole achieving C_max_ after 8 hr incubation time, while carbendazim, iprodione and boscalid attained C_max_ before the first sampling time of 20 min (Fig 2). All of the fungicides achieved the terminal slope after 24 hr incubation time (Fig 2).

In honey, the acaricides, amitraz and bromopropylate, were depleted within 20 min incubation time, while tau-fluvalinate was undetectable after 1hr incubation time (Fig 2). The formation and depletion of amitraz major metabolites, DMF and DMPF, were monitored both in honey and in beeswax for seven consecutive days (Fig 2). Amitraz was completely depleted (spiked amount 5 μg = 0.017 μmol) after 20 min incubation time within the biphasic system, affording 22% DMF (f_m_ = 0.0037 μmol/0.017 μmol) and 30% DMPF (f_m_ = 0.0051 μmol/0.017 μmol) as the total sum in beeswax and honey. DMF was mainly distributed into honey (79%), while DMPF displayed a higher affinity to beeswax (81%). The remaining degradation products of amitraz and its two metabolites constituted 48% of amitraz total mass balance. In honey, DMPF attained its C_max_ value after 24 hr, followed by a monoexponential decline, while DMF achieved C_max_ before the first sampling time point of 20 min, declining subsequently biexponentially (Fig 2). Coumaphos and fenbutatin oxide concentrations in honey were more than 80 and 17 times lower than the corresponding concentrations in beeswax, respectively (Fig 2). Both pesticides attained in honey the terminal slope after 1 hr incubation time. In beeswax, coumaphos and tau-fluvalinate reached their Cmax value before the first sampling time point of 20 min, both of which displayed biexponential decline with a terminal slope, attained after 1 hr incubation time. After 20 min incubation time, more than 80% of both pesticides diffused rapidly into the beeswax phase, followed by 23% total depletion after 7 days incubation time (Fig 2). Bromopropylate and fenbutatin oxide reached in beeswax their C_max_ after 8 hr incubation time, displaying subsequently a biexponential decline, with a terminal slope attained after 24 hr. The C_max_ of DMPF and DMF was achieved after 8 hr incubation time, both of which declined biexponentially and attaining the terminal slope after 24 hr (Fig 2).

**Fig 3:**
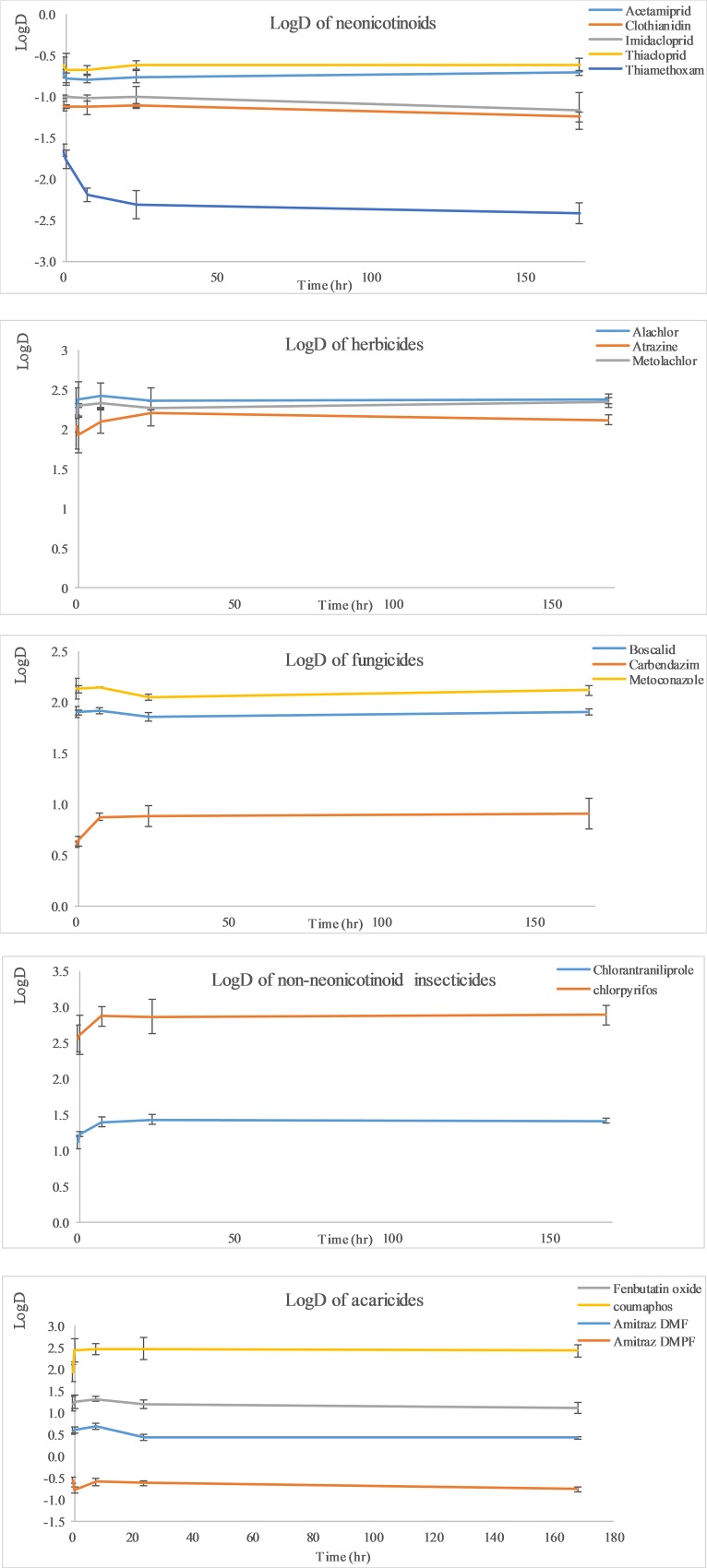
LogD values of selected pesticides, distributed between beeswax and honey as a function of time.

Fig 3 depicts the dependency of LogD values as a function of time. The Fisher test for the linear regression was applied to examine the Null hypothesis of the slope being equal to zero (S3 Table). Accepting the null hypothesis at a significance level of α = 0.05, is a clear indication for the establishment of an equilibrium between pesticide concentrations in beeswax and honey, characterized by a constant ratio of pesticide concentration in beeswax to honey, hence a constant LogD values over time. S3 Table in the supplemental information, provides the Fisher test analysis results together with the time needed to reach a steady state D value. Based on the linear regression F-test, an equilibrium was achieved at the first sampling time point of 0.33 hr, for the neonicotinoids, acetamiprid (Log D = -0.75), clothianidin (Log D = -1.15), imidacloprid (Log D = -1.04) and thiacloprid (Log D = -0.64) (Table 1, Fig 3). Thiamethoxam, however, achieved equilibrium only after 8 hr incubation time, resulting in a fixed LogD value of -2.06 (Table 1, Fig 3). The herbicides, alachlor, metolachlor and atrazine, displayed constant LogD values after 20 min incubation (Table 1). Since residual concentrations of diuron in honey were only available for the first two sampling time points and for oxadiazon and oxyfluorfen, no residual concentrations were detectable at all sampling time points, their corresponding LogD values could not be established.

The LogD values of the fungicides, metconazole and boscalid achieved fixed values of 2.11 and 1.89, respectively within 20 min incubation time (Table 1, Fig 3). Carbendazim, achieved a fixed LogD value of 0.78 after 8 hr incubation time, whereas no LogD values for tetraconazole and iprodione could not be established, due to complete depletion after 20 min in honey (Table 1, Fig 3). Chloranthraniliprole and chlorpyrifos achieved fixed LogD values of 1.32 and 2.75 after 0.33 hr incubation time. Due to complete depletion of cypermethrin and bifenthrin from honey within 20 min, their corresponding LogD values could not be established (Table 1, Fig 3). The acaricides, fenbutatin oxide and coumaphos, as well as the major amitraz metabolites, DMPF and DMF, attained fixed LogD values within 0.33 hr of incubation time (Table 1, Fig 3). For bromopropylate and tau-fluvalinate, LogD values could not be established, due to lack of residual concentrations in honey after 20 min incubation time (Table 1).

### 3.3. Predicting experimentally determined LogD using compound’s descriptors

**Fig 4:**
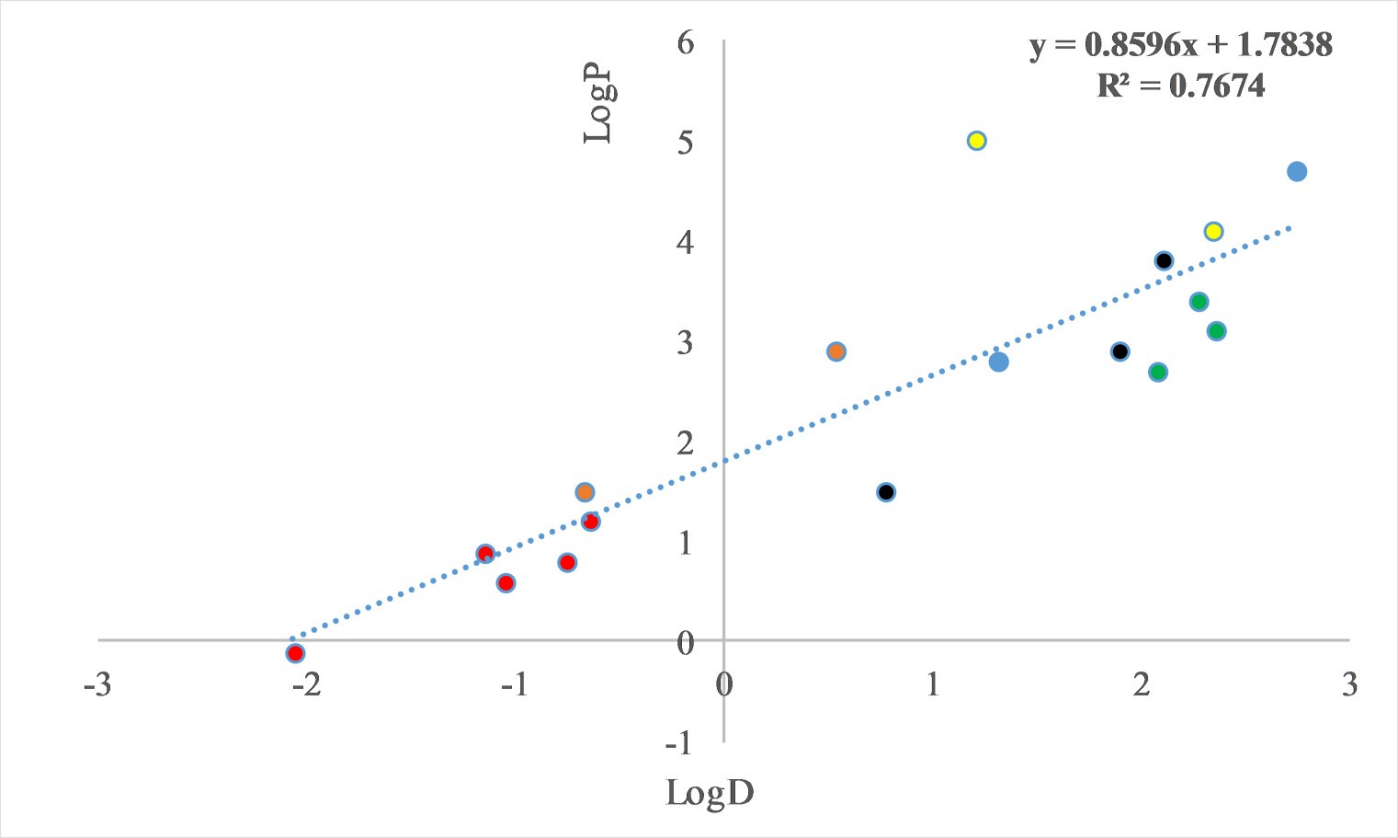
LogD (beeswax/honey distribution) of selected pesticides plotted against LogP (octanol/water partitioning). The pesticide families are denoted as follows: red (neonicotinoids), green (herbicides), blue (non-neonicotinoid insecticides), black (fungicides), yellow (acaricides) and brown (amitraz metabolites, DMF, DMPF).

**Fig 5:**
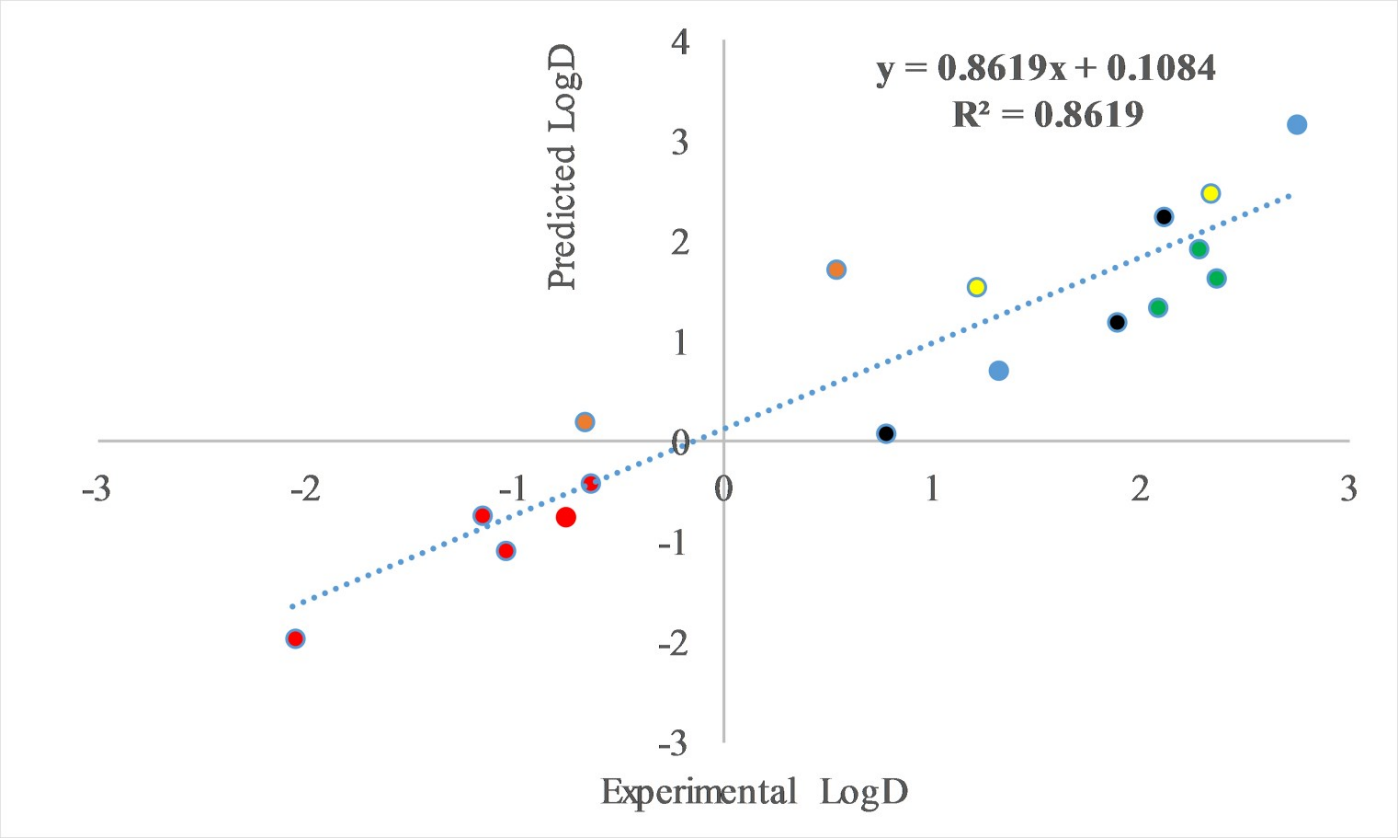
Correlation between experimentally determined LogD values (x-axis) and predicted LogD values (y-axis), obtained from a multiple-linear model: LogD’ = a+b * LogP+c * MW. The proportion of the variance that is predictable from the independent variables was found to be 0.86. The pesticide families are denoted as follows: red (neonicotinoids), green (herbicides), blue (non-neonicotinoid insecticides), black (fungicides), yellow (acaricides) and brown (amitraz metabolites, DMF, DMPF).

For predicting LogD, a set of the descriptors available for the compounds studied was examined (Table 2). This set included LogP and molar weights of pesticides, and the logs of saturated vapor pressures, aqueous solubility and Henry coefficients (provided in Table 2). Multiple regressions of LogD vs. a set of the descriptors were examined. The best-fit single regression was for the LogD values against the corresponding LogP values thus resulting in the linear equation:

LogD = -1.41 (± 0.36) + 0.89 (± 0.13) * LogP (Fig 4). The Fisher F test for linear regression, yielded a significant difference of the linear slope from zero (p = 0.00004) and a R^2^ of 0.77. Including molar weight of pesticides as an additional descriptor into the regression, improved the fitting of the LogD values:

LogD = -1.03 (± 0.31) + 1.10 (± 0.12) * LogP– 0.00276 (± 0.0009) * MW; with an R^2^ value of 0.86 and a significant difference of the linear slope from zero (p = 0.000001). Fig 5 depicts the regression of experimentally determined LogD values against the predicted values, obtained from the model equation, utilizing the two aforementioned substance descriptors. Using any other descriptor (i.e., logs of saturated vapor pressures, aqueous solubility and Henry coefficients) instead of molar weight or adding more than one descriptor did not affect meaningfully the proportion of variance explained.

### 3.4. Pesticide occurrences in beeswax and honey samples collected from Israeli and German beehives

Tables 3 and 4 summarize the mean pesticide concentrations and their prevalence in German beeswax samples (n = 513) and in German (n = 338) and Israeli honey samples (n = 22), respectively. All of the German beeswax samples were contaminated with at least three pesticides occurring simultaneously, while the highest contaminated samples contained up to 27 different pesticides. The following 14 pesticides displayed 100% prevalence in the analyzed beeswax samples: azoxystrobin (n = 23), carbofuran (n = 18), chlorobenzilate (n = 18), cyprodinil (n = 10), dimoxystrobin (n = 35), fenbutatin oxide (n = 35), fenpyroximate (n = 28), fluopyram (n = 17), hexythiazox (n = 28), iprodione (n = 10), metolachlor (n = 10), propargite (n = 49), propoxur (n = 13) and tebuconazole (n = 35) (Table 3). The pesticides with the highest prevalence belonged mainly to the fungicide (43%) and the acaricide class (36%), while the herbicides (1%) and carbamate insecticides (14%) occurred less frequently. Other pesticides revealed high occurrence rate of 85–98% in beeswax at a wide concentration range (Table 3). Cyfluthrin displayed the highest mean pesticide concentration (6.08 mg/kg), followed by iprodione (2.9 mg/kg) and fenvalerate (1.9 mg/kg). In contrast to beeswax, the analysis results of the German honey samples revealed that 75% were free of any pesticide contamination, while the remaining honey samples contained between 1–4 pesticides per sample, at mean pesticide concentrations below the German maximum residue limit (MRL) values, except for clopyralid (Table 4). The mean clopyralid concentration in honey (73 μg/kg) exceeded by a factor of 1.5 the MRL of 50 μg/kg (Table 4). Among the contaminated samples, thiacloprid was the most frequently encountered pesticide with occurrence rate of 46%, followed by clopyralid (34%) and carbendazim (21%). Permethrin displayed the highest mean pesticide concentration in honey (113 μg/kg) followed by clopyralid (73 μg/kg). As for beeswax, the parent compound, amitraz, was absent from all honey samples, while its two major metabolites, DMF and DMPF, occurred only in 8% and 4% of all contaminated honey samples, with mean concentrations 26 μg/kg and 16 μg/kg, respectively (Table 4).

**Table 3:** List of pesticides found in German beeswax samples collected from German apiaries between 2016–2018^a^.

Pesticide	Mean (μg/kg) ± SD^b^ (Range in μg/kg)	% positive^c^ (n)
Acrinathrin	850 ± 2420 (11–10000)	48 (42)
DMF*	110 ± 250 (10–1600)	55 (84)
DMPF*	50 ± 100 (10–300)	12 (59)
Azoxystrobin	20 ± 60 (1–500)	100 (23)
Boscalid	150 ± 680 (1–4300)	90 (40)
Bromopropylate	180 ± 940 (2–10100)	42 (307)
Carbendazim	35 ± 40 (2–100)	95 (21)
Carbofuran	2 ± 1 (0.5–10)	100 (18)
Chlorfenvinphos	200 ± 830 (1–6400)	25 (288)
Chlorobenzilate	30 ± 40 (2–200)	100 (18)
Chlorpyrifos	70 ± 260 (1–1800)	98 (65)
Coumaphos	720 ± 1690 (1–10900)	57 (337)
Cyfluthrin (sum of isomeric mixture)	6080 ± 11200 (400–2300)	19 (21)
λ-Cyhalothrin	690 ± 2260 (3–9100)	48 (33)
Cypermethrin (sum of isomeric mixture)	360 ± 1490 (2–9500)	76 (107)
Cyprodinil	5 ± 3 (2–10)	100 (10)
DDD**	10 ± 10 (2–70)	94 (50)
DDE**	10 ± 10 (1–25)	89 (36)
DDT**	30 ± 40 (2–150)	85 (87)
Deltamethrin	760 ± 2770 (2–11500)	50 (34)
Dimoxystrobin	10 ± 10 (2–30)	100 (35)
Fenbutatin oxide	20 ± 30 (5–80)	100 (10)
Fenpyroximate	40 ± 60 (2–240)	100 (28)
Fenvalerate (sum of 4 optical isomers)	1900 ± 3910 (4–12700)	22 (64)
Flumethrin	160 ± 1050 (1–10900)	35 (310)
Fluopyram	10 ± 4 (2–10)	100 (17)
tau-Fluvalinate,	230 ± 590 (2–4900)	84 (361)
HCH***	20 ± 30 (2–200)	95 (59)
Hexythiazox	10 ± 10 (3–50)	100 (28)
Iprodione	2930 ± 3780 (500–7300)	100 (10)
Malathion	40 ± 110 (1–500)	23 (23)
Metolachlor	3.6 ± 1.5 (2–6)	100 (10)
Permethrin	170 ± 410 (2–2400)	76 (55)
Piperonyl butoxide	50 ± 200 (2–1700)	85 (82)
Propargite	140 ± 250 (2–1000)	100 (49)
Propoxur	10 ± 10 (2–30)	100 (13)
Tebuconazole	10 ± 5.5 (2–20)	100 (35)
Tetramethrin	10 ± 30 (1–130)	71 (55)

^a^Beeswax samples were collected from German apiaries during 2017–2018 and analyzed at the Department of Instrumental Analytic, Bilacon GMbH, Germany. Only pesticides with occurrence concentrations of > 1% were reported herein.

^b^SD, standard deviation.

^c^% positive (n), percentage of positive samples (n = number of samples analyzed).

*DMF, N-(2,4-Dimethylphenyl)-formamide; DMPF, N'-(2,4-Dimethylphenyl)-N-methylformamidine.

**DDD, sum of p,p and p,o-dichlorodiphenyldichloroethane; DDE, sum of p,p- and o,p-dichlorodiphenyldichloroethylene; DDT, dichlorodiphenyltrichloroethane.

***HCH, sum of γ- hexachlorocyclohexane, α- hexachlorocyclohexane, β- hexachlorocyclohexane, and δ- hexachlorocyclohexane isomers

**Table 4:** List of pesticides found in German and Israeli honey samples ^a^.

Pesticide	Mean (μg/kg) ± SD^b^ (Range in μg/kg)	% positive^c^ (N)	Mean (μg/kg) ± SD (Range in μg/kg)	% positive (N)	European MRL^d^ value in honey (μg/kg)	Mean oral LD_50_^e^ after 48 hr (μg/adult bee)	Risk^f^ (%) (German honey)	Risk (%) (Israeli honey)
	Honey from Germany		Honey from Israel					
2,4- Dichloro phenoxyacetate	14 ± 3 (10–18)	9 (64)	2 ± 1 (1–8)	18 (22)	50	94	0.0001	0.00003
Acetamiprid	12 ± 1 (10–13)	10 (39)	2 ±1.0 (1–3)	14 (22)	50	14.3	0.0007	0.0002
DMF*	26 ± 25 (10–89)	8 (162)	24 ±21 (7–90)	96 (22)	200^g^	NA^h^	-^i^	-
DMPF**	18 ± 8 (10–32)	4 (162)	16 ± 15 (6–60)	96 (22)		NA	-	-
Carbendazim	15 ± 8 (10–40)	21 (53)	2.3 ± 2.3 (1–5)	14 (22)	1000	> 756	0.00003	0.000003
Clopyralid	73 ± 58 (12–220)	34 (80)	9 ± 9 (1–21)	23 (22)	50	> 100	0.002	0.0002
Coumaphos	9 ± 10 (1–12)	3 (104)	2 ± 1 (1–4)	96 (22)	100	NA	-	-
Permethrin	113 ± 185 (16–390)	29 (14)	ND^f^	ND	-	0.13	2	-
Thiacloprid	23 ± 22 (10–120)	46 (141)	2 ± 0.7 (1–2)	10 (22)	200	17.3	0.005	0.00009
Imidacloprid	ND^j^	ND	3 ± 1.4 (2–7)	59 (22)	50	0.013	-	1.1
Pirimicarb-desmethyl	ND	ND	2 ± 0.8 (1–3)	27 (22)	-	NA	-	-

^a^German honey samples were collected from German apiaries during January-May 2018 and analyzed at the Department of Instrumental Analytic, Bilacon GMbH, Germany. Israeli honey samples were collected during February-July 2018 from 22 apiaries scattered across the country. Only pesticide, with occurrence concentrations of > 1% were reported herein.

^b^SD, standard deviation.

^c^% positive (n), percentage of positive samples (n = number of samples analyzed).

^d^MRL, maximum residue limit.

^e^LD_50_, mean lethal dose required to kill half the members of a tested population after a specified duration. Data was retrieved from Pesticide Properties Database website: https://sitem.herts.ac.uk/aeru/ppdb/en/

^f^ Risk, was calculated according to the following equation:

% risk = [%] positive samples x average residue dose [μg]/LD50 [μg/adult bee]; the residue dose was obtained from the product, mean residue concentration x average daily intake of honey (80 mg/day) [6].

^g^Amitraz, including the metabolites DMPF, DMF and the 2,4 -dimethylaniline moiety.

^h^NA, data not available.

^i^-, not defined.

^j^ND, not detected.

*DMF, N-(2,4-Dimethylphenyl)-formamide.

**DMPF, N'-(2,4-Dimethylphenyl)-N-methylformamidine.

The organochlorine pesticides, dichlorodiphenyltrichloroethane (DDT) and its two major metabolites, p,p- / p,o-dichlorodiphenyldichloroethane (DDD) and p,p-/ o,p-dichlorodiphenyldichloroethylene (DDE), all of which banned during the early 70s, were found in 85–94% of all German beeswax samples analyzed within the range of 2–150 μg/kg (Table 3). Furthermore, the hexachlorocyclohexane isomers (HCH), γ- HCH, α- HCH, β- HCH, and δ- HCH, which were banned in the European Union in 2008, were found in 95% of all beeswax samples, at a concentration range of 2–200 μg/kg (Table 3).

In contrast to the German honey samples, all of the Israeli honey samples (n = 22) were contaminated with at least two pesticides, among which, 3 samples contained up to 6 pesticides simultaneously. All of the 22 honey samples complied with the European MRL regulations, which were adapted by the Israeli Ministry of Agriculture. The amitraz metabolites, DMF and DMPF, as well as coumaphos were present in 95% of all samples analyzed (Table 4). While the neonicotinoid imidacloprid was absent from all the German honey samples tested during 2018, it was found in 59% of the Israeli honey samples (Table 4). On the other hand, permethrin and carbendazim, found in 29% and 21% of the German samples, were absent from the Israeli honey samples. The mean pesticide concentrations in the Israeli honey samples were generally lower than the corresponding concentrations in the German honey samples (Table 4).

### 3.5. LogD values calculated from pesticide concentrations occurring simultaneously in beeswax and honey samples collected from German beehives

The pesticide residual analysis of German beeswax and honey collected from the same beehives, revealed that only 2 pesticides, namely carbendazim and coumaphos occurred simultaneously in both honey and beeswax phases. The LogD values of carbendazim (0.5) and coumaphos (2.0), calculated from the pesticide concentrations determined in the beehive samples, were very close to those experimentally determined LogD values of 0.78 and 2.3, respectively (Table 1). Due to the low number of co-occurring pesticides in honey and beeswax, a regression analysis was unachievable.

### 3.6. Risk assessment of contaminated honey to bees

Among the pesticides found in German honey samples, only permethrin posed a moderate risk to bee foragers (2%), mostly due to its relatively high mean concentration (113 μg/kg), low LD_50_ (0.13 μg/bee) at a prevalence of 29% (Table 4). The remaining pesticides found in German honey samples posed a negligible dietary risk to bees.

Among the pesticides found in the Israeli honey samples, imidacloprid posed a moderate risk to bee foragers (1%), mainly due to its low mean LD_50_ value (0.013 μg/bee) and its relatively high occurrence rate of 59% (Table 4). The remaining pesticides found in Israeli honey samples posed a negligible dietary risk to bees.

## Discussion

In the present study, our objective was to characterize the persistence and distribution behavior of commonly applied pesticides in beeswax and honey and to relate their kinetic behavior to their occurrence in the field. Since beeswax at room temperature is solid and honey is highly viscous, a free diffusion between beeswax and honey is only feasible at temperatures above the beeswax melting point (65°C) [31]. Beeswax and honey are complex matrices composed of dozens of different compounds, imparting their unique physico-chemical properties [31, 32]. Honey is mostly composed of sugar (about 76%), water (18%) as well as proteins, essential oils, minerals and vitamins [32]. Certain lipophilic compound may solubilize in honey by interacting with solubilized proteins and essential oils [32]. The honey used in the present study, constituted an acidic environment (pH = 3.7–3.4), thereby possibly affecting the ionization state of weak acidic and weak basic pesticides, depending on their respective pKa values and despite the honey’s low water content (Table 1). The ionization state is known to affect substance descriptors such as water solubility, vapor pressure and LogP values [28, 29]. All of the pesticides studied herein were neutral or weak basic pesticides, while for the majority of weak basic pesticides, no public information was available, regarding their respective pKa value and therefore their ionization state could not be estimated. In addition, it is not clear how temperatures as high as 75°C, together with low water content and in the presence of high sugar concentration, affect the pKa value of the tested pesticides. Notwithstanding, we were able to obtain meaningful correlation of LogD with LogP, reflecting the prevalence of pesticides in beeswax and honey samples in the field. Beeswax on the other hand, consists mainly of esters of fatty acids, various long-chain alcohols and hydrocarbons [31]. Hence, partitioning of lipophilic compounds from honey to beeswax is expected primarily for lipophilic pesticides.

The water-soluble neonicotinoids, imidacloprid, acetamiprid, clothianidin, thiacloprid and thiamethoxam displayed slower depletion kinetics in beeswax than in honey, with beeswax terminal half-life being about twice as high as compared to honey (Table 1). Generally, water content is a major contributor to the instability of compounds, due to hydrolytic and oxidative processes [33]. Possibly, neonicotinoids display higher persistence in beeswax than in honey, due to its lower water content and the lack of other molecules such as sugars, which might interact at high temperatures with the pesticides. Since neonicotinoids are more soluble in aqueous media than in hydrophobic solvents, it is not surprising that neonicotinoids display a higher affinity towards honey than towards beeswax (Table 2) [5]. The pesticides half-life determined in the present study, were established under accelerated conditions, namely at 75°C; consequently under field conditions, it is reasonable to expect a significantly longer half-life times for all the pesticides studied herein. Notwithstanding, the half-life times determined in this study, are still of great significance, since they enable us to compare the persistence and honey to beeswax distribution between the pesticides themselves and to relate the results to pesticide molecular properties and eventually to the occurrence in beehive products under field conditions.

The neonicotinoids half-life time in honey of 1.3 to 4.6 days, together with their higher affinity towards honey, render honey as a suitable sentinel for the detection of neonicotinoids applied in vicinity to apiaries (Table 1). Beeswax, however, is less reliable as a bio-indicator for neonicotinoids. Indeed, pesticide survey results support these expectations, clearly demonstrating, that up to about 25% and 95% of the German and Israeli honey samples, respectively, are contaminated with at least one neonicotinoid, while less than 0.1% of beeswax samples were found positive for at least one neonicotinoid (Tables 3 and 4) [18–20].

The herbicides, although belonging to distinct chemical classes (chloroacetamide, triazolinone, phenylureas, oxadiazoles and diphenylethers), share similar values of physico-chemical descriptors, namely LogP values above 2.7, characteristic of their lipophilic nature (Table 2). Except for diuron, all the herbicides demonstrated a greater tendency to distribute into the beeswax phase from honey and displayed significantly longer half-life in beeswax as compared to honey (Table 1). The low water solubility of oxadiazon (0.5 mg/L) and oxyfluorfen (0.1 mg/L), forced both compounds to distribute completely into the beeswax phase, so that no measurable concentrations of the aforementioned compounds were detectable in the honey phase (Fig 2, Table 1). Diuron was the only compound characterized by a zero-order depletion kinetics in beeswax and a rapid depletion in both honey and beeswax phases, resulting in concentrations below the limit of detection (1 μg/kg) in both phases within an hour. The latter observation is in agreement with pesticide survey results in beehive products [6, 10–20].

The fungicides tested, clearly demonstrated a preference to distribute into the beeswax phase, revealing a higher persistence (i.e. longer half-life) in beeswax as compared to the honey phase (Table 1). This observation can be accounted for by their lipophilic nature (LogP values in the range of 1.5–3.8; Table 2). Iprodione and tebuconazole were the only fungicides displaying a complete distribution into the beeswax phase within 20 min distribution time, with no detectable residual concentrations in honey (Fig 2, Table 1). This seems to be reflected also in beehive product surveys, which have reported significant concentrations of both fungicides in beeswax samples and negligible occurrence (and less than 1%) and low residual concentrations in honey samples (Tables 3 and 4) [18–20]. Carbendazim seemed to show a deviation from the other fungicides in terms of half-life in honey and beeswax and its LogD value (Table 1). Carbendazim was characterized by a comparably relatively low LogP value, experimentally demonstrating the lowest LogD value among the fungicides as well as the shortest half-life in both phases (Table 1). The latter observation might be explained by carbendazim pKa value of 4.2, which render it positively charged within the acidic honey environment (pH 3.7–3.4). These properties provided a deeper understanding of carbendazim wide occurrence in both beeswax and honey samples and its relatively low contamination in beehive products (Tables 3 and 4) [6, 10–20]. The LogD values of boscalid and metconazole, namely 1.9 and 2.1, respectively, together with their higher stability in beeswax matrix, provided a scientific explanation for their wide occurrence in beeswax and lower prevalence in honey (Tables 1, 3 and 4) [6, 10–20].

The non-neonicotinoid insecticides, bifenthrin, cypermethrin, chlorpyrifos and chloranthraniliprole constituted the most lipophilic pesticide group, with LogP values ranging between 2.8–6.6 (Table 2). The most lipophilic insecticides tested herein, namely, bifenthrin and cypermethrin, completely distributed into the beeswax phase, resulting in non-detectable concentrations in the honey phase (Fig 2). The half-life value of the latter insecticides in beeswax were amongst the longest experimentally determined half-life (15 and 96 days, respectively; Table 1), an observation also reflected in survey studies, reporting the occasional occurrence of bifenthrin and cypermethrin in beeswax samples, while mostly lacking in honey samples (Tables 3 and 4) [6, 10–20]. In contrast, chloranthraniliprole and chlorpyrifos were found in measurable concentrations in honey over a 7-day distribution period, displaying, on average, honey concentrations 15 and 400 times lower as compared to beeswax (Fig 2). Based on their LogD values of 1.3 and 2.7 as well as their relatively long half-life values of 5.9 and 8.8 days in beeswax, it was expected to find both insecticides mostly in beeswax and rarely in any honey samples analyzed (Table 1). Indeed, our survey results for chlorpyrifos were in agreement with our experimentally derived values, revealing no residual concentrations in honey, while in beeswax, chlorpyrifos was found at high occurrence rate (> 90%) (Tables 3 and 4). Chloranthraniliprole on the other hand, was absent in honey and beeswax samples obtained from German and Israeli apiaries (Tables 3 and 4). This discrepancy may be the result of chloranthraniliprole rapid photo-degradation upon foliage application (8 hr), resulting in undetectable residues being carried by worker bees into the beehive [34]. Moreover, it is reasonable to assume that chloranthraniliprole is completely positively charged within the honey phase, due to its relatively high pKa value of 10.88 (Table 1), explaining its higher honey concentration over time as compared to the other non-neonicotinoid insecticides.

Although, the acaricide class consists of a diverse group of compounds, namely formamidines (amitraz), pyrethroids (tau-fluvalinate), organophosphates (coumaphos), organotins (fenbutatin oxide) and bisbromophenyls (bromopropylate), they all share a common chemical property of being highly lipophilic, with a LogP values ranging between 4.1–7.0 (Table 2). Consequently, it is not surprising that all of them were found ubiquitously at a wide concentration range in beeswax samples, whereas only coumaphos was frequently found at trace concentrations, with the majority of samples complying with the European MRL in honey (100 μg/kg) (Tables 3 and 4). The extensive agricultural and apiary related usage of coumaphos over the years, together with its high persistence and affinity to beeswax (LogD value of 2.34), are considered the main determinants for the widespread coumaphos contamination of beeswax, occasionally even at concentrations up to 10 mg/kg [35]. Kochansky *et al*., showed that coumaphos at a concentration of 100 mg/kg in beeswax was easily transferred into honey upon prolonged contact of several week, reaching honey concentrations of up to 37 μg/kg [35]. This observation may explain the frequent presence of coumaphos in honey at trace concentrations [6, 10–20]. Amitraz is widely used apiary acaricide in Europe and Israel [33]. Due to extremely short half-life, mostly as a result of rapid hydrolytic degradation, amitraz is rarely being detected in honey and beeswax samples, while its two major metabolites, DMF and DMPF, are found to varying degrees in beeswax and honey as indicators of its usage within the beehive [36]. In the present study, the stability and distribution behavior of amitraz was studied in beeswax and honey at 75°C. In the stability study, within 1 min incubation time, amitraz favorably formed in honey the metabolite DMF (fm = 71%), whereas beeswax promoted mostly the formation of the metabolite DMFP (fm = 65%). The distribution outcome for DMPF and DMF complied with the stability study, namely the more lipophilic metabolite DMPF displayed a clear partitioning preference into beeswax (81%; LogD value of 0.5), while the less lipophilic metabolite DMF, showed a higher distribution preference to honey (78%; LogD value of -0.7) (Fig 2, Table 1). In contrast, the German pesticide survey results in honey, revealed lower prevalence of DMF and DMPF, namely 8% and 4%, respectively, possibly indicative of reduced application of amitraz in German apiaries (Table 4). Interestingly, the more hydrophilic amitraz metabolite, DMF, was found in German beeswax samples at a higher prevalence and mean concentration as compared to DMPF, which has been shown to be preferentially formed and partition into beeswax. This apparent contradiction might be resolved, if taking into account DMPF’s shorter half-life in beeswax and honey in comparison to DMF (Table 1).

The longer terminal half-life times of bromopropylate and tau-fluvalinate in beeswax, together with their clear beeswax distribution preference, provide the rational for their relatively high prevalence in beeswax samples, while undetectable in the majority of the analyzed honey samples (Tables 1, 3 and 4). Among the acaricides, fenbutatin oxide is an exceptional compound, due its unusual high molar weight (1052.7 g/mol) and chemical structure (organometallic compound). Fenbutatin oxide was demonstrated to be highly stable in beeswax (t_1/2_ = 32 days) with clear beeswax distribution preference (Table 1). The latter observation is in agreement with fenbutatin oxide high occurrence rate in beeswax samples, while being undetectable in most honey samples analyzed worldwide (Tables 3 and 4) [6, 10–20].

Pesticides undergoing rapid depletion in honey and/or beeswax, a constant LogD value could not be determined, as was the case for bifenthrin, bromopropylate, cypermethrin, diuron, iprodione, oxadiazon, oxyfluorfen, tau-fluvalinate and tebuconazole (Table 1). Except for diuron, which was rapidly eliminated in beeswax, the aforementioned pesticides, rapidly distributed into the beeswax within 20 min incubation time, resulting in undetectable concentrations in honey (Fig 3). For the remaining pesticides listed in Table 1, the conditions allowing obtaining time-independent LogD values were achieved within 20 min to one hr incubation time (Fig 3). As a general rule of thumb, based on the present results, it appears that pesticides with LogD values above one and half-life in beeswax above one day (at 75°C), are likely to accumulate in beeswax and are expected to be found in beeswax samples, and less likely to be found in honey. On the other hand, pesticides with negative LogD values are highly likely to be found in honey and less likely to be encountered in beeswax samples. Finally, pesticides with LogD values between 0–1 are expected to be found in both matrixes (Tables 1, 3 and 4).

Based on the observed study results (half-life and LogD) together with the pesticide occurrence data in honey and beeswax survey studies, we were able to characterize the sentinel capabilities of beeswax and honey towards environmental pesticides. Honey was found to be a suitable sentinel only for hydrophilic pesticides such as neonicotinoids and clopyralid, while beeswax can be of use as a sentinel mostly for highly lipophilic pesticides with LogD values above one. One should take into account, that certain pesticides such coumaphos, tau-fluvalinate and synthetic pyrethroids, exhibit an extraordinary persistence in beeswax and consequently tend to bio-accumulate, potentially yielding concentration values of dozens of mg/kg per sample. For such compounds, beeswax may be less suitable as a sentinel for environmental exposure, due to a masking of a newly emerged contamination by the high pesticide background. As a general guideline and for pesticides being monitored only once a year, compounds with half-lives less than 90 days in beeswax (at beehive temperature of 34°C) are more suitable candidates for biomonitoring, since after 4 half-life, more than 90% of the compound is eliminated [26]. Hence, the pesticides half-life in relation to the sampling frequency and the distribution tendency of each pesticide within the beehive compartments are major determinants of pesticide prevalence in honey and beeswax in survey studies, providing the rational for their wide occurrence variability and the lack of detectability of numerous pesticides in honey and/or beeswax samples. Consequently, the sentinel definition of each beehive matrix is limited only to certain pesticide classes, so that looking for highly lipophilic pesticides in honey or highly hydrophilic pesticides in beeswax would be redundant, especially if their corresponding half-lives are very short. Moreover, the quantification of pesticide residues in various matrixes is a function of the sensitivity and specificity of the analytical method employed. Hence, it is likely that more sensitive analytical methods would be able to detect hydrophilic pesticides such as neonicotinoids in lipophilic matrixes such as beeswax. Therefore, the hereby-proposed rule of thumb depends upon the method sensitivity and therefore is only valid for the analytical method utilized in the present study.

Since beeswax is a constituent of commercially available comb honey and is also frequently used as food additive (E 901), in the preparation of pastries, as a postharvest treatment as well as a flavor carrier, it is proposed to implement action limit for the presence of residues in beeswax [11]. Currently, in the USA and Europe, there are no legal requirements regarding the regulation of pesticide residues in beeswax and any other beehive product except for honey [37]. Since beeswax was demonstrated in many studies, to be highly contaminated with multiple pesticides and other environmental pollutants, it would be more than reasonable to suggest the implementation of MRL values for beeswax used for human consumption [11]. The latter is also proposed for pollen, which is widely used in the cosmetic, nutraceutical and food supplement industry [38–40]. Furthermore, to the best of our knowledge, MRL’s for pesticides or other chemical residues are presently not specifically defined in Europe and the USA, for food additives, cosmetic ingredients and nutraceuticals.

Pollen and beebread are widely accepted as generally more reliable sentinels for assessing the presence of environmental pesticides and pesticide contamination of beehives, respectively [38, 39]. In contrast to beeswax and honey, a wider range of pesticides of various physico-chemical properties have been found in pollen and beebread, thereby bearing the advantage of covering pesticides found only in beeswax or in honey samples [38, 39]. Within the beehive, pollen are being processed and stored in the form of beebread, a complex non-homogenous mixture of pollen, honey, bee secretions and microorganisms [40]. Consequently, a simple partitioning of pesticides between beebread and honey is actually impractical. However, adsorption studies of pesticides to pollen, dispersed within honey, are feasible and worthwhile carrying out. The latter suggested adsorption experiment will enable us to identify and characterize pesticides, which can be bio-monitored by bee pollen analysis. The role of the *n*-octanol/water partition coefficient for organic compounds has become increasingly important in predicting environmental fate [41]. Consequently, we have examined the correlation between LogD to LogP for all the pesticides studied, in order to evaluate the predictive power of LogP. It is important to note, that both parameters were determined at different temperatures, namely LogD was measured at 75°C, while LogP is a value determined at room temperature [27]. The excellent correlation between the LogD and LogP values, suggests that the obtained experimental LogD values can be used to predict LogP values, despite the experimental temperature differences.

The ANOVA regression analysis results indicated a significant correlation between the two parameters (p = 0.000004) yielding an R^2^ value of 0.77. Notably, the slope of the regression line was 0.89 (±0.13), i.e., not differing significantly from one (Fig 4). This suggests that, differences in pesticide interactions in *n*-octanol and water, expressed as LogP value and pesticide distribution between beeswax and honey, were similarly affected by changes in pesticide molecular structure. Moreover, the obtained results propose that, *n*-octanol-water partitioning may indeed mimic pesticide interactions in complicated matrices as beeswax and honey and might be useful for predicting pesticide distribution between different compartments within the beehive [38]. Fenbutatin oxide was the only pesticide, displaying the largest deviation from the linear regression (Fig 4). The removal of fenbutatin oxide, resulted in a substantial improvement of the linear correlation, yielding an R^2^ of 0.95. Fenbutatin oxide large MW (1052.7 g /mol) distinguishes it from the rest of the pesticides, while the remaining pesticides exhibit a MW range of 142.2–483.2 g/mol (Table 2). The MW may affect the kinetics of partitioning from honey to beeswax, as the rate of diffusion is inversely proportional to the square root of molar mass [42]. Hence, it is reasonable to assume, that the large MW, associated with small diffusion coefficients, may lead to underestimation of LogD values. Therefore, using MW as an additional descriptor improved the extent of association between LogD and LogP, yielding an R^2^ value of 0.86 (Fig 5). The intercepts of the different models varied between -1.25 to -0.78, indicative of a generally reduced extent of distribution from honey to beeswax as compared to water-to-octanol distribution. The correlation between experimentally determined and predicted LogD values (Fig 5) differentiates neonicotinoids from other pesticides. Since all of the neonicotinoids tested hereby are highly water soluble, they displayed a pronounced distribution preference into the honey phase, resulting in negative LogD values. The other pesticides that are more hydrophobic displayed a greater distribution tendency into the beeswax phase, yielding positive LogD values. Consequently, the neonicotinoid’s negative LogD values enabled their clear distinction from the other pesticides within the correlation analysis (Fig 5). The pesticide risk assessment in the present study was focused on honey, as pesticides trapped within beeswax are less likely to affect bees and their larvae [6]. The only two pesticides found in the German and Israeli honey, namely permethrin and imidacloprid, respectively, constituted a moderate risk to honeybees wellbeing (Table 4), primarily due to their low mean oral LD_50_ values and relatively high occurrence rate of 29% and 59%, respectively (Table 4). Since the honey consumption of worker larvae is about one third of the honey consumption of forager bee and for a shorter time period (~ 5 days), the calculated risk of dietary exposure to imidacloprid and permethrin in honey was 0.4% and 0.7%, respectively, hence regarded as low [6]. Notwithstanding, the present risk assessment simplifies real field conditions, since honeybees are exposed to multiple pesticides during their entire life span, which are commonly found in pollen, surrounding atmosphere and drinking water [43].

## Conclusion

The present study provided a quantitative scientific foundation for understanding and predicting the distribution and accumulation potential of various pesticides in beeswax and honey, based on their LogD and half-life values. We have established a mathematical expression, enabling us to predict the LogD of each pesticide, thereby providing us the necessary knowledge to characterize the sentinel spectrum of honey and beeswax towards organic pesticides. Based on the multi-linear regression model, we have found that LogP and the MW, were the most prominent parameters affecting LogD values, and consequently the distribution of pesticides between honey and beeswax within beehives. Beeswax is a major contamination sink of environmental pollutants, thereby constituting hazardous health implications for bees as well as for humans. Due to beeswax extensive usage in the pharmaceutical, nutraceutical and cosmetic industry, decontamination procedures are warranted and regulatory agencies are calling for considering the establishment of MRL values for beeswax [6].

## Supporting information

S1 Table MRM transition and optimized parameters for pesticides analyzed by LC-MS/MS. (PDF)

S2 Table MRM transition and optimized parameters for the pesticides analyzed by GC-MS/MS with positive EI ionization. (PDF)

S3 Table Linear regression F-test of LogD vs time. (PDF)

## References

[1] Ollerton J, Winfree R, Tarrant S. How many flowering plants are pollinated by animals? Oikos 2011; 120: 321–326.

[2] Nazzi F, Pennacchio F. Disentangling multiple interactions in the hive ecosystem. Trends Parasitol. 2014; 30: 556–561. 10.1016/j.pt.2014.09.006 25457396

[3] Mitchell EAD, Mulhauser B, Mulot M, Mutabazi A, Glauser G, Aebi A. A worldwide survey of neonicotinoids in honey. Science 2017; 358: 109–111. 10.1126/science.aan3684 28983052

[4] Krupke CH, Hunt GJ, Eitzer BD, Andino G, Given K. Multiple routes of pesticide exposure for honey bees living near agricultural fields. PLoS One 2012; 7: e29268 10.1371/journal.pone.0029268 22235278 PMC3250423

[5] Niell S, Jesús F, Pérez N, Pérez C, Pareja L, Abbate S, et al Neonicotinoids transference from the field to the hive by honey bees: Towards a pesticide residues biomonitor. Sci Total Environ. 2017; 581–582: 25–31. 10.1016/j.scitotenv.2017.01.011 28073057

[6] Sanchez-Bayo F, Goka K. Pesticide residue and bees–a risk assessment. PLoS One. 2014; 9: e94482 10.1371/journal.pone.0094482 24718419 PMC3981812

[7] Iyer M, Mishra R, Han Y, Hopfinger AJ. Predicting blood-brain barrier partitioning of organic molecules using membrane-interaction QSAR analysis. Pharm Res. 2002; 19: 1611–1621. 12458666 10.1023/a:1020792909928

[8] Arnot JA, Gobas FA. A food web bioaccumulation model for organic chemicals in aquatic ecosystems. Environ Toxicol Chem. 2004; 23: 2343–2355. 15511097 10.1897/03-438

[9] Goss KU, Schwarzenbach RP. Linear free energy relationships used to evaluate equilibrium partitioning of organic compounds. Environ Sci Technol. 2001; 35: 1–9. 11351988 10.1021/es000996d

[10] Perugini M, Tulini SMR, Zezza D, Fenucci S, Conte A, Amorena M. Occurrence of agrochemical residues in beeswax samples collected in Italy during 2013–2015. Sci Total Environ. 2018; 625: 470–476. 10.1016/j.scitotenv.2017.12.321 29291561

[11] Wilmart O, Legrève A, Scippo ML, Reybroeck W, Urbain B, de Graaf DC, et al Residues in Beeswax: A Health Risk for the Consumer of Honey and Beeswax? J Agric Food Chem. 2016; 64: 8425–8434. 10.1021/acs.jafc.6b02813 27741395

[12] Niell S, Jesús F, Pérez C, Mendoza Y, Díaz R, Franco J, et al QuEChERS adaptability for the analysis of pesticide residues in beehive products seeking the development of an agroecosystem sustainability monitor. J Agric Food Chem. 2015; 63: 4484–4492. 10.1021/acs.jafc.5b00795 25880394

[13] Calatayud-Vernich P, Calatayud F, Simó E, Picó Y. Occurrence of pesticide residues in Spanish beeswax. Sci Total Environ. 2017; 605–606: 745–754. 10.1016/j.scitotenv.2017.06.174 28679118

[14] Chiesa LM, Panseri S, Nobile M, Ceriani F, Arioli F. Distribution of POPs, pesticides and antibiotic residues in organic honeys from different production areas. Food Addit Contam Part A Chem Anal Control Expo Risk Assess. 2018; 35:1340–1355. 10.1080/19440049.2018.1451660 29533158

[15] Al-Alam J, Fajloun Z, Chbani A, Millet M. A multiresidue method for the analysis of 90 pesticides, 16 PAHs, and 22 PCBs in honey using QuEChERS-SPME. Anal Bioanal Chem. 2017; 409: 5157–5169. 10.1007/s00216-017-0463-y 28681076

[16] Boi M, Serra G, Colombo R, Lodesani M, Massi S, Costa C. A 10 year survey of acaricide residues in beeswax analyzed in Italy. Pest Manag Sci. 2016; 72:1366–1372. 10.1002/ps.4161 26423556

[17] Anastassiades M, Lehotay SJ, Stajnbaher D, Schenck FJ. Fast and easy multiresidue method employing acetonitrile extraction/partitioning and "dispersive solid-phase extraction" for the determination of pesticide residues in produce. J AOAC Int. 2003; 86: 412–431. 12723926

[18] Amulen DR, Spanoghe P, Houbraken M, Tamale A, de Graaf DC, Cross P, et al Environmental contaminants of honeybee products in Uganda detected using LC-MS/MS and GC-ECD. PLoS One. 2017; 12: e0178546 10.1371/journal.pone.0178546 28570581 PMC5453540

[19] Chauzat MP, Martel AC, Cougoule N, Porta P, Lachaize J, Zeggane S, et al An assessment of honeybee colony matrices, Apis mellifera (Hymenoptera: Apidae) to monitor pesticide presence in continental France. Environ Toxicol Chem. 2011; 30: 103–111. 10.1002/etc.361 20853451

[20] Valdovinos-Flores C, Alcantar-Rosales VM, Gaspar-Ramírez O, Saldaña-Loza LM, Dorantes-Ugalde JA. Agricultural pesticide residues in honey and wax combs from Southeastern, Central and Northeastern Mexico. J Apicultural Res. 2017; 56: 667–679.

[21] European Commission, 2011. Commission Implementing Regulation (Eu) No 540/2011of 25 May 2011.

[22] Kah M, Beulke S, Brown CD. Factors influencing degradation of pesticides in soil. J Agric Food Chem. 2007; 55: 4487–4492. 10.1021/jf0635356 17488087

[23] Farha W, Abd El-Aty AM, Rahman MM, Shin HC, Shim JH. An overview on common aspects influencing the dissipation pattern of pesticides: a review. Environ Monit Assess. 2016; 188: 693–714. 10.1007/s10661-016-5709-1 27888425

[24] Jan J, Cerne K. Distribution of some organochlorine compounds (PCB, CBZ, and DDE) in beeswax and honey. Bull Environ Contam Toxicol. 1993; 51: 640–646. 8241618 10.1007/BF00201639

[25] Wolt JD, Nelson HP Jr, Cleveland CB, van Wesenbeeck IJ. Biodegradation kinetics for pesticide exposure assessment. Rev Environ Contam Toxicol. 2001; 169:123–164. 11330076 10.1007/978-1-4613-0107-3_2

[26] Gibaldi M, Perrier D. Pharmacokinetics. 2nd ed Marcel Dekker, INC; New York 1982.

[27] OECD (1995). Test No. 107: Partition Coefficient (n-octanol/water): Shake Flask Method, OECD Guidelines for the Testing of Chemicals, Section 1, OECD Publishing, Paris, 10.1787/9789264069626-en.

[28] Tülp HC1, Goss KU, Schwarzenbach RP, Fenner K. Experimental determination of LSER parameters for a set of 76 diverse pesticides and pharmaceuticals. Environ Sci Technol. 2008; 42: 2034–2040. 18409633 10.1021/es702473f

[29] Jin X, Fu Z, Li X, Chen J. Development of polyparameter linear free energy relationship models for octanol-air partition coefficients of diverse chemicals. Environ Sci Process Impacts. 2017; 19: 300–306. 10.1039/c6em00626d 28154864

[30] Rortais A, Arnold G, Halm MP, Touffet-Briens F. Modes of honeybees exposure to systemic insecticides: estimated amounts of contaminated pollen and nectar consumed by different categories of bees. Apidologie 2005; 36: 71–83.

[31] Aichholz R, Lorbeer E. Investigation of comb wax of honeybees with high temperature gas chromatography and high-temperature gas chromatography-chemical ionisation mass spectrometry. J Chromatogr. 1999; 855: 601–615. 10.1016/s0021-9673(99)00725-6 10519097

[32] da Silva PM, Gauche C, Gonzaga LV, Costa AC, Fett R. Honey: Chemical composition, stability and authenticity. Food Chem. 2016; 196: 309–323. 10.1016/j.foodchem.2015.09.051 26593496

[33] Coats JR. Pesticide degradation mechanisms and environmental activation. ACS Symposium Series 1991; 459: 10–30.

[34] Sharma AK, Zimmerman WT, Singles SK, Malekani K, Swain S, Ryan D, et al Photolysis of chlorantraniliprole and cyantraniliprole in water and soil: verification of degradation pathways via kinetics modeling. J Agric Food Chem. 2014; 62: 6577–6584. 10.1021/jf5017175 24971760

[35] Kochansky J, Wilzer K, Feldlaufer M. Comparison of the transfer of coumaphos from beeswax into syrup and honey. Apidologie 2001; 32:119–125.

[36] Korta E, Bakkali A, Berrueta LA, Gallo B, Vicente F, Kilchenmann V, et al Study of acaricide stability in honey. Characterization of amitraz degradation products in honey and beeswax. J Agric Food Chem. 2001; 49: 5835–5842. 11743771 10.1021/jf010787s

[37] European Commission. Summary report of the standing committee on the food chain and animal health held in Brussels on 13 June 2013–14 June 2013.

[38] Tosi S, Costa C, Vesco U, Quaglia G, Guido G. A 3-year survey of Italian honey bee-collected pollen reveals widespread contamination by agricultural pesticides. Sci Total Environ. 2018; 615: 208–218. 10.1016/j.scitotenv.2017.09.226 28968582

[39] de Oliveira RC, Queiroz SCDN, da Luz CFP, Porto RS, Rath S. Bee pollen as a bioindicator of environmental pesticide contamination. Chemosphere. 2016; 163: 525–534. 10.1016/j.chemosphere.2016.08.022 27567152

[40] Anderson KE, Carroll MJ, Sheehan T, Lanan MC, Mott BM, Maes P, et al Hive-stored pollen of honey bees: many lines of evidence are consistent with pollen preservation, not nutrient conversion. Mol Ecol. 2014; 23: 5904–5917. 10.1111/mec.12966 25319366 PMC4285803

[41] Finizio A, Vighi M, Sandroni D. Determination of n-octanol/water partition coefficient (Kow) of pesticide critical review and comparison of methods. Chemosphere 1997; 34: 131–161.

[42] Laidler KJ, Meiser JH. Physical Chemistry Menlo Park, Calif: Benjamin/Cummings Pub. Co, 1982; pp. 18–19.

[43] Majewski MS, Capel PD. Pesticides in the atmosphere: distribution, trends, and governing factors. U.S. geological survey, open-file report, 1995; pp. 94–506

