# Supplementary material for: Pesticide distribution and depletion kinetic determination in honey and beeswax: Model for pesticide occurrence and distribution in beehive products
Source: PLoS One. 2019 Feb 20;14(2):e0212631. doi: 10.1371/journal.pone.0212631 (PMC6382162; doi:10.1371/journal.pone.0212631)
Supplement: S1 Table — (PDF) [file pone.0212631.s001.pdf]

S1 Table. MRM transition and optimized parameters for pesticides analyzed by LC-MS/MS.

A. Positive ESI

Compound	DP (v)	EP (v)	CE (v)	CXP (v)	Q1 (g/mol)	Q2 (g/mol)	Retention Time (min)
(Internal Standard) Bifenthrin D6	37.99	10	27.1	12	312.8	163.9	10.51
(Internal Standard) Triphenylphosphat	37.7	10	27.1	12	312.8	163.7	8.41
4-Bromophenylurea (Metabolite Metobromuron)	37.41	10	27.1	12	312.8	163.4	4.81
Acephat	37.12	10	27.1	12	312.9	163.2	1.01
Acequinocyl	36.83	10	27.1	12	312.9	163.0	12.01
Acetamiprid	36.55	10	27.1	12	313.0	162.7	4.01
Acetochlor	36.26	10	27.1	11.9	313.0	162.5	7.60
Acibenzolar-S-methyl	35.97	10	27.1	11.9	313.0	162.3	7.130
Aclonifen	35.68	10	27.1	11.9	313.1	162.1	8.01
Acrinathrin	35.39	10	27.1	11.9	313.1	161.8	10.01
Alachlor	35.1	10	27.1	11.9	313.2	161.6	7.81
Aldicarb	34.82	10	27.1	11.9	313.2	161.4	4.51
Aldicarb-sulfoxid	34.53	10	27.1	11.9	313.2	161.1	1.160
Aldoxycarb (Aldicarb-sulfuron)	34.24	10	27.1	11.9	313.3	160.9	1.240
Allethrin	33.95	10	27.1	11.9	313.3	160.7	9.11
Ametoctradin	33.66	10	27.1	11.9	313.4	160.5	8.61
Ametryn	33.38	10	27.1	11.9	313.4	160.2	7.11
Amidosulfuron	33.09	10	27.1	11.9	313.5	160.0	2.51
Aminocarb	32.8	10	27.1	11.9	313.5	159.8	5.51
Aminopyralid	32.51	10	27.1	11.9	313.5	159.6	1.01
Amisulbrom	32.22	10	27.1	11.9	313.6	159.3	8.91
Amitraz	31.93	10	27.1	11.9	313.6	159.1	9.81
Amitraz Metabolite DMA (2,4-Dimethylanilin)	31.65	10	27.1	11.8	313.7	158.9	4.08
Amitraz Metabolite DMF	31.36	10	27.1	11.8	313.7	158.6	5.01
Amitraz Metabolite DMPF	31.07	10	27.1	11.8	313.7	158.4	0.00
Atrazin	30.49	10	27.1	11.8	313.8	158.0	6.20
Atrazin-desethyl	29.63	10	27.1	11.8	313.9	157.3	4.0
AvermectinB1a	29.34	10	27.1	11.8	314.0	157.0	10.01
AvermectinB1b	29.05	10	27.1	11.8	314.0	156.8	9.91
Azaconazol	28.76	10	27.1	11.8	314.1	156.6	6.61
Azadirachtin	28.47	10	27.1	11.8	314.1	156.4	5.10
Azamethiophos	28.19	10	27.1	11.8	314.1	156.1	5.50
Azinphos-ethyl	27.9	10	27.1	11.8	314.2	155.9	7.70
Azinphos-methyl 3	27.61	10	27.1	11.8	314.2	155.7	7.00
Azinphos-methyl 4	27.32	10	27.1	11.8	314.3	155.4	7.00
Azoxystrobin	27.03	10	27.1	11.7	314.3	155.2	7.10
BAC C 18	26.75	10	27.1	11.7	314.3	155.0	8.51
BAC C 20	26.46	10	27.1	11.7	314.4	154.8	9.150
BAC C 22	26.17	10	27.1	11.7	314.4	154.5	9.220

Benalaxyl	25.88	10	27.1	11.7	314.5	154.3	8.31
Bendiocarb	25.59	10	27.1	11.7	314.5	154.1	5.01
Benfuracarb	25.3	10	27.1	11.7	314.5	153.8	9.01
Benodanil	25.02	10	27.1	11.7	314.6	153.6	6.31
Benoxacor	24.73	10	27.1	11.7	314.6	153.4	7.01
Bensulfuron-methyl	24.44	10	27.1	11.7	314.7	153.2	6.71
Benthiavalicarb Isopropyl	24.15	10	27.1	11.7	314.7	152.9	7.51
Benzylaminopurin	23.86	10	27.1	11.7	314.7	152.7	5.21
Demeton-S-methyl-sulfon	41	10	23	12	263.2	168.9	2.10
Desmedipham	21	10	19	14	318.3	182.2	6.80
Diafenthiuron	86	10	27	10	385.3	329.0	9.00
Dialifos	41	10	23	12	395.8	187.0	8.60
Diazinon	19	10	29	8	305.2	169.0	8.40
Dichlofenthion	26	10	21	14	314.9	258.9	9.20
Dichlofluanid	24	8.5	21	12	350.0	223.9	7.70
Dichlorbenzamid 2	76	10	77	14	191.9	74.0	2.50
Dichlorvos	26	10	27	6	221.0	108.9	5.40
Diclobutrazol	56	10	49	12	328.1	70.0	8.00
Diclofop-Me	26	10	21	16	358.0	281.0	9.00
Dicrotophos	41	10	19	8	238.1	112.1	3.00
Diethofencarb	29	10	15	12	268.1	226.1	7.10
Diethyltoluamid (DEET)	51	10	43	12	192.1	90.9	6.40
Difenoconazol	81	10	35	18	406.0	250.9	8.50
Difenoxuron	39	10	25	6	287.1	123.1	6.60
Difenoxuron 2	39	10	25	6	287.1	72.1	6.50
Diflufenican	81	10	33	16	395.0	266.0	8.70
Dimefuron	61	10	29	8	339.1	167.0	6.90
Dimethachlor	66	10	19	14	256.1	223.9	7.00
Dimethenamid	41	10	21	16	275.9	243.7	7.20
Dimethoat	31	10	15	14	230.1	198.9	3.60
Dimethomorph	41	10	27	18	388.1	301.1	7.10
Dimoxystrobin	56	10	15	14	327.1	205.0	8.00
Diniconazol	54	10	47	12	326.6	70.1	8.40
Dinotefuran	51	10	17	24	203.1	129.1	1.00
Dioxacarb	91	10	11	14	223.9	167.0	3.10
Dioxathion	16	10	11	16	474.1	271.1	9.00
Diphenamid	81	10	29	24	240.2	134.1	6.70
Dipropetryn	61	10	39	12	256.2	144.1	8.10
Disulfoton	9	10	17	4	275.1	89.1	8.50
Disulfoton-Sulfon	51	10	41	16	307.1	96.9	6.40
Disulfoton-Sulfoxid	31	10	19	12	291.1	184.7	6.30
Ditalimfos	51	10	27	10	300.1	148.0	7.70
Diuron	41	10	39	6	233.2	72.2	6.50
DMST	41	10	19	8	215.2	106.0	5.60
Dodemorph	86	10	29	8	282.4	116.1	10.00
Dodin 1	101	10	37	4	228.3	60.1	8.40

Dodin 2	101	10	27	14	228.3	186.0	8.40
DTAC	76	10	43	10	229.3	60.1	8.00
Edifenphos	81	10	19	12	311.0	283.0	8.10
Emamectin B1a	26	10	121	12	886.2	82.1	9.30
Emamectin B1b	56	10	117	10	872.2	82.0	9.10
EPN	51	10	19	18	324.1	296.0	8.40
Epoxiconazol	51	10	95	14	330.2	75.0	7.80
EPTC	71	10	15	12	190.0	128.0	8.00
Ethiofencarb	31	10	21	8	226.2	107.1	6.00
Ethiofencarb 2	31	10	11	14	226.2	169.1	5.50
Ethiofencarb sulfon	11	10	31	10	275.3	107.0	3.00
Ethiofencarb-sulfoxide	21	10	29	10	242.3	107.1	3.00
Ethion	20	10	17	10	385.5	199.1	9.20
Ethirimol	46	10	31	8	210.2	140.0	6.30
Ethofumesate	24	10	27	6	304.1	121.1	7.10
Ethoprophos	26	10	29	6	243.3	131.1	7.70
Ethoxyquin	81	10	43	8	218.2	160.2	7.70
Etofenprox	26	10	19	12	394.2	177.2	10.40
Etoxazol	56	10	79	6	360.2	113.0	9.50
Etrimfos	56	10	25	22	293.0	264.9	8.20
Famoxadon	14	10	23	12	392.2	238.8	8.30
Famphur	66	10	33	6	326.0	125.0	6.50
Fenamidon	44	10	19	12	312.1	92.2	7.20
Fenamiphos	46	10	31	12	304.2	217.0	7.90
Fenamiphos-Sulfon	71	10	23	8	336.1	308.1	5.80
Fenamiphos-Sulfoxid	81	10	23	8	320.1	292.0	5.60
Fenarimol	96	10	49	14	331.2	139.0	7.70
Fenazaquin	36	10	31	8	307.2	161.2	9.80
Fenbuconazol	41	10	37	6	337.1	125.1	7.80
Fenbutatinoxid	111	10	35	12	519.3	463.2	12.00
Fenchlorazol-ethyl	86	10	33	10	403.8	357.8	8.40
Fenhexamid	121	10	31	10	301.9	97.1	7.60
Fenitrothion	61	10	31	8	278.1	125.0	7.50
Fenobucarb	41	10	13	8	208.1	152.0	7.00
Fenoxycarb	36	10	31	8	302.4	87.9	7.90
Fenpiclonil	10	10	35	10	253.9	202.1	6.80
Fenpropathrin	41	10	19	10	350.2	125.1	9.60
Fenpropidin	86	10	41	14	274.3	147.1	7.20
Fenpropimorph	41	10	43	14	304.3	146.9	10.00
Fenpyrazamin	61	10	45	36	332.2	189.1	7.60
Fenpyroximat	21	10	23	20	422.2	366.3	9.70
Fensulfothion	56	10	21	16	309.1	281.1	6.60
Fensulfothion-oxon	61	10	25	22	293.0	236.8	4.70
Fensulfothion-oxon-sulfon	71	10	21	18	309.2	253.0	5.00
Fensulfothion-sulfon	71	10	23	18	325.0	268.7	6.80
Fenthion	31	10	23	8	279.1	169.1	8.10

Fenthion-Oxon	71	10	23	16	262.7	230.9	6.80
Fenthion-Oxon-Sulfon	91	10	29	20	295.2	217.0	4.00
Fenthion-Oxon-Sulfoxid	71	10	39	18	279.2	104.0	3.80
Fenthion-Sulfon	71	10	31	8	311.1	125.0	6.10
Fenthion-Sulfoxid	81	10	45	20	295.1	109.1	5.90
Fentin	96	10	33	14	351.2	196.7	0.00
Fenuron	21	10	27	10	165.1	72.2	3.50
Fenvalerat	36	10	21	16	437.0	167.0	9.90
Fipronil	14	10	29	20	453.9	368.1	8.00
Flamprop-Methyl	61	10	19	12	335.9	104.9	7.50
Flazasulfuron	41	10	25	4	408.1	182.3	4.80
Flonicamid	61	10	25	12	230.2	203.1	2.50
Florasulam	51	10	31	12	359.9	128.9	3.50
Fluazifop free acid	61	10	27	16	328.2	282.1	5.80
Fluazifop-p-butyl	51	10	29	10	384.1	281.7	9.00
Flucythrinat	36	10	45	10	469.2	181.0	9.50
Fluensulfon	86	10	25	10	291.8	165.9	6.50
Flufenacet	51	10	17	18	364.1	194.1	7.70
Flufenoxuron	56	10	25	12	489.1	158.0	9.50
Flufenzin	46	10	21	12	305.0	138.0	8.00
Flumethrin	36	10	21	22	526.9	266.9	10.30
Flumioxazin	101	10	29	8	355.1	327.2	7.00
Fluopicolid	61	10	31	14	385.0	175.0	7.50
Fluopyram	81	10	43	16	397.1	173.0	7.70
Fluoxastrobin	96	10	25	12	459.1	427.2	7.70
Fluoxypyr-meptyl	116	10	15	20	369.0	256.9	9.40
Flupyradifuron	106	10	29	10	291.1	128.0	3.90
Fluquinconazol	54	10	25	20	376.0	349.0	7.60
Flurochloridon	91	10	31	6	314.0	294.0	7.60
Flusilazol	36	10	25	14	316.1	247.1	7.90
Fluthiacet-Me	191	10	31	10	405.9	346.1	7.50
Flutolanil	71	10	27	26	324.2	262.1	7.40
Flutriafol	44	10	39	6	302.1	122.9	6.40
Fluxapyroxad	66	10	21	8	382.2	362.1	7.40
Fomesafen	46	10	21	4	456.1	344.0	6.70
Forchlorfenuron	56	10	25	8	248.0	128.9	6.40
Formetanat	46	10	23	10	222.2	165.1	0.00
Formothion	41	10	11	12	258.1	198.8	5.10
Fosthiazat	61	10	27	4	284.0	104.1	6.10
Fuberidazol	26	10	29	32	185.1	157.1	5.30
Furametapyr	66	10	45	28	334.2	157.0	6.30
Furathiocarb	46	10	25	20	383.2	194.9	9.00
Halfenprox	44	10	27	10	494.1	183.1	10.80
Halosulfuron-methyl	66	10	31	10	435.0	182.0	5.00
Haloxyfop-2-ethoxyethyl	41	10	29	12	434.2	316.1	8.90
Haloxyfop-methyl	56	10	25	10	376.6	316.1	8.70

Heptenophos	31	10	19	6	251.1	127.0	6.60
Hexaconazol	31	10	39	4	314.1	70.1	8.20
Hexazianon	61	10	25	16	253.1	171.1	5.50
Hexythiazox	41	10	21	12	353.2	228.1	9.20
Icaridin (Picaridin)	86	10	21	24	230.2	130.1	6.70
Imazalil	41	10	31	4	297.2	159.0	8.10
Imazamox	106	10	29	24	306.0	261.0	2.10
Imazaquin	51	10	37	10	312.2	199.1	3.00
Imazethaphyr	51	10	37	10	290.1	177.2	2.50
Imazosulfuron	71	10	19	10	412.9	153.0	4.70
Imidacloprid	36	10	23	16	256.1	209.5	3.00
Indoxacarb	51	10	21	10	528.1	293.1	8.70
Iodosulforonmethyl	41	10	29	12	508.1	167.1	4.80
Iprobenfos	66	10	31	6	288.9	91.0	8.00
Iprodion	31	10	21	10	330.1	245.0	7.80
Iprovalicarb	21	10	31	14	321.3	119.1	7.70
Isazophos	66	10	37	8	314.0	120.0	7.60
Isofenphos	51	10	17	18	346.2	244.9	8.50
Isofenphos-Methyl	26	10	9	16	332.2	273.3	8.10
Isofenphos-Oxon	36	10	17	6	329.8	229.0	7.60
Isomethiozin	26	10	17	12	269.2	200.1	8.20
Isoprocarb	41	10	21	6	194.1	95.1	6.30
Isoprothiolane	41	10	17	42	291.0	231.1	7.40
Isoproturon	41	10	31	14	207.3	71.9	6.40
Isopyrazam	66	10	23	8	360.3	340.2	8.50
Isoxaben	66	10	17	12	333.1	85.0	7.30
Isoxaflutole	21	10	27	14	377.1	251.0	6.10
Isoxathion 2	44	10	21	6	314.0	105.0	8.50
Ivermectin B1a	71	10	37	18	892.6	307.0	10.80
Ivermectin B1b	66	10	35	16	878.5	293.2	10.50
Kresoxim-Methyl	14	10	11	14	314.1	205.9	8.00
Landrin (3,4,5-Trimethacarb)	31	10	17	10	194.4	137.1	6.50
Lenacil	26	10	23	8	235.2	153.1	6.30
Linuron	36	10	25	16	249.2	159.7	7.00
Malaoxon	31	10	17	6	315.5	127.1	5.70
Malathion	24	10	17	6	331.1	127.0	7.50
Mandestrobin	71	10	15	12	314.2	192.1	8.10
Mandipropamid	76	10	21	10	412.2	328.1	7.30
Mecabarm 1	19	10	15	12	330.0	227.0	7.80
Mecabarm 2	61	10	55	18	330.0	97.0	7.70
Mefenpyr-diethyl	71	10	47	10	375.1	161.9	8.30
Mepanipyrim	51	10	37	10	224.2	106.0	7.60
Mepronil	21	10	39	12	270.2	119.1	7.40
Mesosulfuron-methyl	61	10	33	12	504.1	181.9	5.50
Metalaxyl	41	10	19	12	280.3	220.0	6.50
Metamitron	36	10	25	12	203.2	174.9	3.50

Metazachlor	10	10	15	10	278.1	210.1	6.50
Metconazol	96	10	59	10	320.1	69.9	8.30
Methabenzthiazuron	11	10	43	8	222.1	150.0	6.20
Methacrifos	26	10	11	12	241.1	209.0	6.90
Methamidophos	66	10	19	14	142.1	94.0	1.00
Methidathion	21	10	15	6	302.9	145.1	6.90
Methiocarb	31	10	15	12	226.3	169.9	7.10
Methiocarbsulfon	29	10	29	6	275.1	122.0	4.20
Methiocarbsulfoxid	36	10	19	36	242.2	185.1	3.50
Methomyl	21	10	13	8	163.2	88.0	2.30
Methothrin	51	10	25	10	321.2	135.1	8.00
Methoxyfenozid	39	10	23	8	369.2	149.1	7.60
Metobromuron	61	10	29	16	261.0	172.0	6.20
Metolachlor	26	10	19	10	284.2	252.2	7.90
Metolcarb	86	10	17	8	166.0	109.1	5.00
Metosulam	86	10	35	20	419.0	174.9	5.50
Metoxuron	61	10	39	12	229.2	72.0	4.80
Metrafenon	61	10	21	12	409.1	209.0	8.50
Metribuzin	29	10	25	10	215.1	187.2	5.40
Metsulfuron-methyl	36	10	31	14	382.1	198.8	3.10
Mevinphos	29	10	13	10	225.0	193.1	4.60
MGK 264	101	10	19	14	276.1	210.1	8.70
Milbemectin A3	86	10	11	20	546.1	511.2	9.80
Milbemectin A4	96	10	11	20	560.0	525.2	9.60
Molinat	21	10	19	6	188.1	126.1	7.40
Monalide	76	10	29	16	239.9	85.1	7.90
Monocrotophos	31	10	23	10	224.2	126.9	2.40
Monolinuron	61	10	37	14	215.2	126.0	6.00
Monuron	56	10	23	2	199.1	72.0	5.20
Myclobutanil	46	10	39	12	289.2	70.0	7.40
Napropamid	56	10	23	8	272.2	129.1	7.70
Napthylessigsaeureamid	66	10	23	12	186.0	141.1	5.00
Nicosulfuron	46	10	31	12	411.1	182.1	6.70
Nitenpyram	31	10	35	6	271.2	126.0	2.00
Nitralin	36	10	21	16	346.2	304.1	7.88
Nurarimol	44	10	43	4	315.0	81.1	7.10
Omethoat	36	10	19	12	214.3	183.0	1.30
Oxadiazon	26	10	25	16	362.2	303.3	9.10
Oxadixyl	36	10	13	14	279.2	219.0	5.00
Oxamyl	1	10	19	6	237.3	71.8	1.80
Oxamyl-oxime	36	10	21	12	162.9	72.3	1.00
Oxydemeton-Methyl	36	10	21	14	247.2	169.0	2.00
Oxyfluorfen	41	10	27	6	379.2	316.0	9.00
Paclobutrazol	66	10	41	12	294.1	70.1	7.30
Paraoxon-Ethyl	74	10	19	12	276.1	220.0	6.40
Paraoxon-Methyl	54	10	35	6	248.0	109.1	5.10

Parathion_Methyl	76	10	25	12	263.9	125.0	7.10
Parathion-Ethyl	49	10	21	14	292.0	236.1	8.10
Penconazol	41	10	39	8	284.1	158.9	8.00
Pencycuron	46	10	33	6	329.1	125.1	8.50
Pendimethalin	6	10	15	10	282.1	212.2	9.30
Penthiopyrad	101	10	21	18	360.0	276.0	8.10
Permethrin	41	10	13	10	408.2	355.1	10.20
Pethoxamid	56	10	29	24	296.0	131.0	7.70
Phenmedipham	41	10	13	4	301.3	168.0	7.00
Phenothrin	71	10	29	10	351.3	183.0	10.10
Phenthoate	36	10	17	14	321.1	247.0	8.10
Phorat, -sulfon	71	10	15	12	292.9	171.0	6.50
Phorat, -sulfoxid	76	10	13	14	277.2	198.8	6.20
Phorate	13	11	18	8	261.1	75.0	8.40
Phosalone	51	10	21	10	367.9	182.0	8.40
Phosmet 1	26	10	15	10	317.9	159.8	7.00
Phosmet 2	26	10	57	10	317.9	132.9	7.00
Phosmet-oxon	71	10	19	8	302.1	159.9	5.00
Phoxim	61	10	17	22	299.0	128.9	8.40
Picoxystrobin	81	10	13	12	368.2	205.0	8.00
Pinoxaden	131	10	29	14	400.9	317.1	8.40
Piperonylbutoxid	21	10	17	12	356.1	177.1	9.10
Piperophos	181	10	31	12	353.8	171.0	8.70
Pirimicarb	16	10	29	10	239.3	71.9	6.20
Pirimicarb-desmethyl	36	10	31	6	225.3	71.7	5.00
Pirimicarb-desmethyl-formamido	31	10	29	4	253.2	71.7	5.50
Pirimiphos-Ethyl	21	10	29	10	334.1	198.1	9.20
Pirimiphos-Methyl	24	10	29	8	306.1	164.1	8.50
Prallethrin	96	10	17	8	301.1	133.1	8.60
Primisulfuron-methyl	46	10	29	12	469.1	254.0	6.30
Prochloraz	24	10	17	16	376.0	308.0	8.40
Prochloraz-desimidazol-amino	71	10	23	28	327.1	284.0	8.20
Prochloraz-desimidazol-formylamino	66	10	21	18	355.0	310.1	8.20
Profenofos	46	10	25	18	372.9	302.9	8.80
Promecarb	36	10	13	12	208.3	150.8	7.10
Prometryn	46	10	33	8	242.2	158.1	7.70
Propamocarb	16	10	25	6	189.4	101.9	0.00
Propaquizafop	66	10	31	6	444.2	100.0	9.00
Propargit	4	10	21	8	368.1	175.1	9.50
Propazin	51	10	25	12	230.2	188.1	7.00
Propetamophos	26	10	23	8	282.2	138.0	7.50
Propham	11	10	13	10	180.3	138.2	6.30
Propiconazol	46	10	33	4	342.1	69.1	8.20
Propisochlor	56	10	13	14	283.9	224.0	8.20
Propoxur	31	10	21	10	210.3	111.1	5.50
Propoxycarbazone sodium	51	10	19	12	421.0	180.1	0.00

Propyzamid	39	10	31	8	256.0	173.1	7.40
Proquinazid	66	10	21	10	373.0	330.8	9.60
Prosulfocarb	36	10	29	4	252.1	91.2	8.80
Prosulfuron	46	10	31	14	420.2	141.0	5.80
Prothioconazol-Desthio	61	10	47	12	312.1	70.0	7.80
Prothiofos	46	10	25	22	344.9	240.9	9.90
Pymetrozin	31	10	31	10	218.3	105.0	2.00
Pyraclostrobin	66	10	17	10	388.2	194.1	8.30
Pyraflufen-ethyl	91	10	29	8	415.0	341.0	8.30
Pyrazophos	61	10	29	12	374.1	222.1	8.50
Pyrazosulfuron-ethyl	46	10	23	12	415.2	182.0	5.50
Pyrethrin I	66	10	13	10	329.1	160.8	9.50
Pyrethrin II	76	10	15	10	373.0	160.8	8.70
Pyridaben	31	10	19	16	365.2	309.1	9.80
Pyridalyl	61	10	41	20	492.0	110.9	10.90
Pyridaphenthion	76	10	31	12	341.1	188.9	7.50
Pyridat (Metabolit)	36	10	29	12	207.0	104.0	2.50
Pyridate	36	10	29	12	379.2	207.0	10.50
Pyrifenox	16	10	31	4	295.0	93.1	7.80
Pyrimethanil	41	10	35	10	200.2	107.0	7.00
Pyrimidifen	96	10	33	34	378.2	184.1	9.10
Pyriproxifen	14	10	21	4	322.1	96.2	9.10
Quinalphos	36	10	29	12	299.1	97.1	8.10
Quinclorac	66	10	55	12	242.1	161.1	2.40
Quinmerac	26	10	21	12	222.2	203.8	2.00
Quinoxyfen	21	10	57	8	307.9	162.0	9.20
Quizalofop free acid	66	10	27	16	345.2	299.1	6.50
Quizalofop-ethyl	61	10	29	14	373.0	299.0	8.90
Quizalofop-p-tefuryl	146	10	29	18	429.0	299.0	8.80
Resmethrin	76	10	21	12	339.3	171.0	10.00
Rimsulfuron	51	10	29	14	432.1	182.0	3.50
Rotenone	71	10	33	12	395.1	213.1	7.90
Saflufenacil	96	10	61	10	501.2	198.0	6.40
Sebuthylazine	61	10	25	10	230.3	174.0	7.00
Sedaxan	111	10	17	16	332.1	312.0	7.50
Sethoxydim	41	10	29	14	328.3	177.9	8.30
Simazin	66	10	37	6	202.2	132.1	5.50
Spinetoram J	96	10	47	14	748.4	142.2	10.00
Spinetoram L	101	10	43	26	760.5	142.2	10.30
Spinosyn A	61	10	41	10	732.3	141.9	9.70
Spinosyn D	61	10	45	14	746.5	142.0	10.00
Spirodiclofen	151	10	29	8	411.0	71.1	9.70
Spirotetramat	61	10	25	6	374.3	302.2	7.70
Spirotetramat-cis-Keto-Hydroxy	71	10	37	12	318.2	214.1	6.50
Spirotetramat-Enol	96	10	39	20	302.2	216.1	5.50
Spirotetramat-Enol-Glucosid 1	56	10	21	8	464.3	302.2	3.60

Spirotetramat-Enol-Glucosid 2	56	10	63	12	464.3	216.1	3.60
Spirotetramat-Mono-Hydroxy	71	10	27	14	304.2	254.1	5.40
Spiroxamine	51	10	29	12	298.4	144.1	7.80
Sulcotrione	51	10	25	8	329.1	138.7	1.50
Sulfosulfuron	31	10	19	12	471.1	211.0	4.20
Sulfotep	41	10	21	10	323.1	171.0	8.20
tau-Fluvalinat	46	10	47	14	520.1	181.0	10.10
tau-Fluvalinat 2	31	10	23	4	520.1	208.1	10.10
Tebuconazol	51	10	37	6	308.2	69.7	8.10
Tebufenozid	21	10	13	10	353.3	297.2	8.00
Tebufenpyrad	101	10	49	10	334.1	117.2	9.00
Tebutam	16	10	33	16	234.2	91.1	7.90
Tebuthiuron	66	10	25	12	229.0	171.9	5.60
Tembotrion	96	10	15	8	441.1	341.1	4.60
Tepraloxydim	56	10	19	14	342.1	250.1	6.30
Terbufos	21	10	13	18	289.1	103.0	9.00
Terbufossulfon	36	10	23	10	339.7	171.1	7.10
Terbufossulfoxid	61	10	17	10	304.9	186.9	7.30
Terbumeton	36	10	25	10	226.2	170.1	7.10
Terbuthylazin	51	10	25	12	230.1	173.9	7.20
Terbutryn	41	10	27	10	242.2	186.1	7.70
Tetrachlorvinphos	51	10	21	6	367.0	127.0	8.00
Tetraconazol	81	10	43	10	372.2	158.9	7.70
Tetramethrin	21	10	29	10	349.3	164.1	8.80
Thiabendazol	56	10	37	12	202.1	175.0	5.00
Thiabendazol-5-hydroxy	66	10	35	14	218.2	191.0	3.80
Thiacloprid	41	10	29	8	253.1	125.7	4.40
Thiamethoxam	36	10	17	18	292.2	211.1	2.00
Thifensulfuron-methyl	36	10	21	18	387.9	167.0	3.00
Thiobencarb	56	10	67	14	258.0	88.9	8.40
Thiocyclam	51	10	23	8	182.0	136.7	4.70
Thiodicarb	31	10	31	8	355.0	88.0	6.20
Thiofanox	11	10	15	4	219.5	57.1	5.90
Thiofanox sulfon	61	10	25	10	251.1	57.0	3.70
Thiofanox sulfoxid	16	10	19	10	252.2	103.8	3.00
Thiophanat-methyl	36	10	37	4	343.0	150.8	5.50
Tolclofos-Methyl	44	10	23	16	301.0	268.9	8.50
Tolfenpyrad	71	10	39	12	384.1	197.1	9.00
Tolylfluanid	9	10	19	12	364.0	237.9	8.50
Tralkoxydim	51	10	29	26	330.2	138.1	7.40
Triadimefon	36	10	21	10	294.0	197.2	7.50
Triadimenol	36	10	31	12	296.1	69.9	7.50
Triadimenol 2	36	10	13	14	296.1	227.0	7.50
Triasulfuron	46	10	29	12	402.1	167.0	4.60
Triazamat	34	10	33	10	315.1	72.1	7.70
Triazophos	31	10	47	6	314.1	119.1	7.50

Triazophos 2	61	10	29	12	314.0	162.1	7.50
Triazoxide	86	10	53	12	248.0	68.0	6.60
Tribenuron-methyl	46	10	21	8	396.1	155.0	5.30
Trichlorfon	46	10	33	18	274.0	109.0	3.70
Tricyclazol	91	10	33	30	190.1	163.0	4.70
Tridemorph	96	10	37	10	298.4	130.2	10.50
Trifloxystrobin	21	10	23	10	409.1	186.1	8.70
Triflumizol	6	10	17	10	346.0	278.0	8.70
Triflumizol-Metabolit	66	10	47	6	295.0	43.4	7.50
Triflusulfuron-methyl	46	10	31	12	493.1	264.1	6.60
Triforin	26	10	15	10	435.0	389.8	6.80
Triticonazol	96	10	21	8	318.1	70.0	7.60
Tritosulfuron	81	10	29	12	446.1	195.0	5.50
Valiphenal	81	10	53	8	399.1	155.1	7.50
Vamidothion	26	10	19	12	288.2	145.8	3.50
Zoxamid	65	10	35	4	336.2	187.1	8.20

B. Negative ESI

Compound	DP (v)	EP (v)	CE (v)	CXP (v)	Q1 (g/mol)	Q2 (g/mol)	Retention Time (min)
CAP (Internal Standard)	-65.0	-10.0	-24.0	-11.0	321.0	152.1	3.50
2- Naphtoxyacetic acid 1	-105.0	-10.0	-18.0	-13.0	200.9	142.9	3.80
2 -Naphtoxyacetic acid 2	-105.0	-10.0	-50.0	-11.0	200.9	115.0	3.80
2,4-D 1	-19.0	-10.0	-14.0	-10.0	219.0	160.9	3.80
2,4-D 2	-19.0	-10.0	-34.0	-26.0	219.0	124.9	3.80
2,4-DB 1	-64.0	-10.0	-12.0	-10.0	247.0	160.8	3.90
2,4-DB 2	-64.0	-10.0	-34.0	-10.0	247.0	124.9	3.90
2-Naphtylacetic acid	-50.0	-10.0	-11.0	-12.0	185.0	140.8	3.80
4-CPA 1	-71.0	-10.0	-18.0	-10.0	185.0	126.8	3.70
4-CPA 2	-71.0	-10.0	-12.0	-26.0	185.0	140.8	3.70
Bentazon 1	-56.0	-10.0	-32.0	-10.0	239.1	132.0	3.70
Bentazon 2	-56.0	-10.0	-24.0	-12.0	239.1	197.0	3.70
Bromoxynyl 1	-51.0	-10.0	-36.0	-14.0	273.9	79.0	3.80
Bromoxynyl 2	-51.0	-10.0	-40.0	-12.0	277.9	80.9	3.80
Chlorfluazuron 1	-66.0	-10.0	-30.0	-14.0	538.0	517.9	4.40
Chlorfluazuron 2	-54.0	-10.0	-28.0	-12.0	538.0	354.9	4.40
Clethodim	-54.0	-10.0	-14.0	-14.0	358.1	238.1	4.00
Cyantranilirpol 1	-80.0	-10.0	-18.0	-11.0	472.7	203.8	3.70
Cyantranilirpol 2	-80.0	-10.0	-18.0	-13.0	472.7	201.8	3.70
Cyclanilid 1	-65.0	-10.0	-28.0	-11.0	271.9	159.9	4.20
Cyclanilid 2	-65.0	-10.0	-16.0	-15.0	271.9	227.9	4.20
Cycloxydim 1	-61.0	-10.0	-68.0	-12.0	324.2	235.9	4.20
Cycloxydim 2	-61.0	-10.0	-68.0	-12.0	324.2	133.8	4.20
Dicamba 1	-21.0	-10.0	-6.0	-12.0	219.0	175.0	3.80
Dicamba 2	-21.0	-10.0	-14.0	-10.0	219.0	144.8	3.80

Dichloran 1	-66.0	-10.0	-20.0	-7.0	205.0	175.0	4.00
Dichloran 2	-66.0	-10.0	-24.0	-7.0	205.0	168.9	4.00
Dichlorprop 1	-26.0	-10.0	-14.0	-12.0	233.0	161.0	3.90
Dichlorprop 2	-26.0	-10.0	-34.0	-26.0	233.0	125.1	3.90
Diflubenzuron 1	-31.0	-10.0	-12.0	-7.0	308.9	288.9	4.00
Diflubenzuron 2	-31.0	-10.0	-14.0	-7.0	308.9	155.9	4.00
Diflubenzuron 3	-31.0	-10.0	-68.0	-7.0	308.9	92.9	4.00
Dinocap 1	-95.0	-10.0	-42.0	-13.0	295.0	208.7	4.50
Dinocap 2	-95.0	-10.0	-72.0	-7.0	295.0	133.7	4.50
Dinoseb 1	-36.0	-10.0	-52.0	-14.0	239.1	134.0	4.20
Dinoseb 2	-36.0	-10.0	-30.0	-12.0	239.1	193.0	4.20
Dinoterb 1	-49.0	-10.0	-24.0	-16.0	239.1	207.0	4.20
Dinoterb 2	-49.0	-10.0	-24.0	-14.0	239.1	176.0	4.20
Dithianon 1	-24.0	-10.0	-24.0	-10.0	296.0	264.0	4.00
Dithianon 2	-24.0	-10.0	-28.0	-20.0	296.0	238.0	4.00
DNOC 1	-26.0	-10.0	-24.0	-18.0	197.0	137.0	3.90
DNOC 2	-26.0	-10.0	-28.0	-6.0	197.0	108.8	3.90
Endosulfansulfat 1	-69.0	-10.0	-38.0	-18.0	420.8	96.8	4.10
Endosulfansulfat 2	-69.0	-10.0	-130.0	-6.0	420.8	79.9	4.10
Fipronil 1	-34.0	-10.0	-18.0	-22.0	435.0	330.0	4.10
Fipronil 2	-34.0	-10.0	-38.0	-18.0	435.0	250.0	4.10
Fipronil-Desulfinyl 1	-54.0	-10.0	-34.0	-16.0	387.0	351.0	4.10
Fipronil-Desulfinyl 2	-54.0	-10.0	-16.0	-24.0	387.0	282.0	4.10
Fipronil-Sulfid 1	-16.0	-10.0	-34.0	-18.0	419.0	262.0	4.10
Fipronil-Sulfid 2	-16.0	-10.0	-20.0	-26.0	419.0	383.0	4.10
Fipronil-Sulfon 1	-24.0	-10.0	-18.0	-16.0	451.0	282.0	4.20
Fipronil-Sulfon 2	-24.0	-10.0	-50.0	-8.0	451.0	415.0	4.20
Fluazifop free acid 1	-31.0	-10.0	-18.0	-26.0	326.1	253.8	3.90
Fluazifop free acid 2	-31.0	-10.0	-50.0	-24.0	326.1	108.1	3.90
Fluazinam 1	-56.0	-10.0	-40.0	-9.0	462.9	415.8	4.30
Fluazinam 2	-56.0	-10.0	-40.0	-7.0	462.9	397.9	4.30
Flubendiamid 1	-90.0	-10.0	-40.0	-1.0	681.0	253.9	4.00
Flubendiamid 2	-90.0	-10.0	-24.0	-13.0	681.0	273.9	4.00
Fludioxonil 1	-56.0	-10.0	-44.0	-9.0	247.0	179.9	3.90
Fludioxonil 2	-56.0	-10.0	-54.0	-11.0	247.0	126.0	3.90
Gibberilic Acid 1	-90.0	-10.0	-46.0	-7.0	344.9	142.7	3.20
Gibberilic Acid 2	-90.0	-10.0	-22.0	-15.0	344.9	238.9	3.20
Haloxyfop free acid 1	-90.0	-10.0	-22.0	-19.0	359.8	287.8	4.00
Haloxyfop free acid 2	-90.0	-10.0	-54.0	-11.0	359.8	195.7	4.00
Haloxyfop free acid 4	-90.0	-10.0	-36.0	-13.0	359.8	251.8	4.00
Hexaflumuron 1	-41.0	-10.0	-26.0	-7.0	458.9	438.8	4.20
Hexaflumuron 3	-41.0	-10.0	-26.0	-9.0	458.9	275.7	4.20
Ioxynil 1	-46.0	-10.0	-36.0	-10.0	369.8	126.8	3.80
Ioxynil 2	-46.0	-10.0	-26.0	-14.0	369.8	214.9	3.80
Iprodion	-74.0	-10.0	-20.0	-10.0	328.0	141.1	4.20
Lufenuron 1	-90.0	-10.0	-16.0	-29.0	510.7	340.9	4.30

Lufenuron 2	-90.0	-10.0	-50.0	-15.0	510.7	176.8	4.30
MCPA 1	-29.0	-10.0	-10.0	-10.0	198.9	140.8	3.80
MCPA 2	-55.0	-10.0	-40.0	-9.0	198.8	104.9	3.80
MCPA 3	-140.0	-10.0	-40.0	-15.0	201.0	143.0	3.80
MCPB 1	-51.0	-10.0	-14.0	-26.0	227.0	140.9	4.00
MCPB 2	-26.0	-10.0	-36.0	-1.0	227.0	104.8	4.00
Mecoprop-P 1	-51.0	-10.0	-22.0	-9.0	213.0	140.7	3.90
Mecoprop-P 2	-51.0	-10.0	-14.0	-9.0	213.0	71.0	3.90
Meptyldinocap 1	-20.0	-10.0	-40.0	-19.0	295.0	193.0	4.50
Meptyldinocap 2	-20.0	-10.0	-34.0	-19.0	295.0	193.9	4.50
Metaflumizone 1	-150.0	-10.0	-24.0	-13.0	505.0	302.1	4.30
Metaflumizone 2	-150.0	-10.0	-74.0	-19.0	505.0	285.0	4.30
Methoxyfenozid	-66.0	-10.0	-30.0	-26.0	367.2	148.8	3.80
Novaluron 1	-55.0	-10.0	-18.0	-21.0	490.9	471.0	4.20
Novaluron 2	-55.0	-10.0	-20.0	-15.0	490.9	305.0	4.20
Picloram 1	-60.0	-10.0	-14.0	-21.0	240.8	196.8	3.40
Picloram 2	-60.0	-10.0	-30.0	-9.0	240.8	122.8	3.40
Prohexadion 1	-21.0	-10.0	-16.0	-18.0	211.1	123.1	3.60
Prohexadion 2	-21.0	-10.0	-16.0	-46.0	211.1	166.9	3.60
Prothioconazol 1	-80.0	-10.0	-32.0	-7.0	343.9	99.9	4.00
Prothioconazol 2	-80.0	-10.0	-84.0	-1.0	343.9	57.9	4.00
Quizalifop 1	-54.0	-10.0	-30.0	-18.0	343.1	270.9	4.00
Quizalifop 2	-54.0	-10.0	-44.0	-12.0	343.1	242.9	4.00
Sulfentrazon 1	-100.0	-10.0	-34.0	-15.0	385.0	307.0	3.70
Sulfentrazon 2	-100.0	-10.0	-52.0	-9.0	385.0	198.9	3.70
Teflubenzuron 1	-26.0	-10.0	-10.0	-9.0	379.1	338.7	4.30
Teflubenzuron 2	-26.0	-10.0	-18.0	-7.0	379.1	195.9	4.30
TFNA 1	-45.0	-10.0	-16.0	-7.0	189.8	145.7	3.40
TFNA 2	-45.0	-10.0	-46.0	-11.0	189.8	68.9	3.40
TFNA 3	-45.0	-10.0	-36.0	-23.0	189.8	126.0	3.40
TFNG 1	-60.0	-10.0	-18.0	-11.0	246.8	182.7	3.10
TFNG 2	-60.0	-10.0	-26.0	-9.0	246.8	162.7	3.10
TFNG 3	-60.0	-10.0	-32.0	-9.0	246.8	134.8	3.10
Triclopyr 1	-50.0	-10.0	-16.0	-11.0	253.9	195.7	3.80
Triclopyr 2	-50.0	-10.0	-8.0	-11.0	253.9	217.9	3.80
Triflumuron 1	-50.0	-10.0	-18.0	-6.0	357.0	153.9	4.10
Triflumuron 2	-50.0	-10.0	-52.0	-6.0	357.0	85.3	4.10
Trinexapac-ethyl	-75.0	-10.0	-28.0	-7.0	251.0	136.8	3.80
Trinexapac-ethyl 2	-75.0	-10.0	-26.0	-7.0	251.0	132.9	3.80
Halosulfuron-Me 1	-75.0	-10.0	-26.0	-1.0	433.0	251.9	4.10
Halosulfuron-Me 2	-75.0	-10.0	-38.0	-7.0	433.0	154.0	4.10
Fomesafen 1 e	-95.0	-10.0	-50.0	-9.0	437.0	194.9	4.10
Fomesafen 2 e	-95.0	-10.0	-44.0	-11.0	437.0	221.8	4.10
Metamitron 1	-75.0	-10.0	-28.0	-9.0	201.0	117.1	3.30
Metamitron 2	-75.0	-10.0	-18.0	-11.0	201.0	185.0	3.30
Azadirachtin 1	-105.0	-10.0	-22.0	-5.0	719.2	485.1	3.70

Azadirachtin 2	-110.0	-10.0	-52.0	-6.0	719.2	99.0	3.70
Acephat 1	-75.0	-10.0	-16.0	-9.0	181.9	140.9	0.00
Acephat 2	-75.0	-10.0	-30.0	-7.0	181.9	79.0	0.00
Sulfoxaflor 1	-70.0	-10.0	-22.0	-11.0	275.9	211.7	3.40
Sulfoxaflor 2	-70.0	-10.0	-22.0	-9.0	275.9	212.9	3.40
Saflufenacil 1	-105.0	-10.0	-52.0	-17.0	499.1	328.0	3.80
Saflufenacil 2	-105.0	-10.0	-40.0	-3.0	499.1	347.8	3.80
Aminopyralid 2	-50.0	-10.0	-14.0	-7.0	204.9	160.8	1.30
Aminopyralid 3	-50.0	-10.0	-14.0	-7.0	206.9	162.9	1.30
Diuron 1	-65.0	-10.0	-32.0	-9.0	231.0	149.8	3.80
Diuron 2	-65.0	-10.0	-24.0	-9.0	231.0	185.8	3.80
Daminozid 1	-45.0	-10.0	-14.0	-7.0	159.0	140.9	1.20
Daminozid 2	-45.0	-10.0	-20.0	-5.0	159.0	96.8	1.20
Sedaxan 1	-90.0	-10.0	-36.0	-5.0	330.0	111.0	3.80
Sedaxan 2	-90.0	-10.0	-30.0	-5.0	330.0	131.0	3.80
Dalapon 1	-30.0	-10.0	-10.0	-10.0	141.0	105.0	3.40
Dalapon 2	-30.0	-10.0	-12.0	-12.0	141.0	97.0	3.40
Dichlorophen 1	-100.0	-10.0	-30.0	-9.0	266.8	127.0	3.90
Dichlorophen 2	-100.0	-10.0	-84.0	-15.0	266.8	35.0	3.90
Chlorbufam 1	-40.0	-10.0	-10.0	-9.0	221.9	177.9	3.80
Chlorbufam 2	-40.0	-10.0	-12.0	-7.0	221.9	175.9	3.80
Acifluorfen 1	-45.0	-10.0	-22.0	-7.0	359.9	285.5	4.20
Acifluorfen 2	-45.0	-10.0	-12.0	-9.0	359.9	315.8	4.20
Fluoxypyr 1	-50.0	-10.0	-10.0	-1.0	252.9	232.8	3.60
Fluoxypyr 2	-50.0	-10.0	-20.0	-9.0	252.9	194.8	3.60
Imazamox 1	-90.0	-10.0	-10.0	-18.0	304.0	260.1	3.40
Imazamox 2	-90.0	-10.0	-44.0	-13.0	304.0	186.0	3.40
Asulam 1	-50.0	-10.0	-22.0	-19.0	228.9	196.8	2.90
Asulam 2	-50.0	-10.0	-26.0	-9.0	228.9	105.8	2.90
Acibenzolar acid 1	-50.0	-10.0	-28.0	-5.0	178.8	106.8	3.70
Acibenzolar acid 2	-50.0	-10.0	-16.0	-7.0	178.8	134.9	3.70
Fenoprop 1	-50.0	-10.0	-40.0	-22.0	267.0	159.0	3.90
Fenoprop 2	-50.0	-10.0	-40.0	-22.0	267.0	195.0	3.90
2-4-5-T 1	-50.0	-10.0	-40.0	-22.0	253.0	159.0	3.90
2-4-5-T 2	-50.0	-10.0	-40.0	-22.0	253.0	195.0	3.90
Quinclorac 1	-30.0	-10.0	-10.0	-11.0	239.9	195.9	3.60
Mesotrione 1	-50.0	-10.0	-14.0	-19.0	338.1	290.9	3.60
Mesotrione 2	-50.0	-10.0	-44.0	-15.0	338.1	212.0	3.60
(Internal Standard) Diuron D6	-135.0	-10.0	-26.0	-13.0	236.9	185.8	3.80
Benzovindiflupyr 1	-165.0	-10.0	-30.0	-9.0	398.0	369.9	4.00
Benzovindiflupyr 2	-165.0	-10.0	-66.0	-13.0	398.0	90.9	4.00
Bromacil 1	-95.0	-10.0	-28.0	-11.0	259.0	202.9	3.70
Bromacil 2	-95.0	-10.0	-46.0	-1.0	259.0	78.9	3.70
Bentazon-6-OH 1	-100.0	-10.0	-26.0	-17.0	254.9	190.8	3.60
Bentazon-6-OH 2	-100.0	-10.0	-34.0	-17.0	254.9	147.8	3.60
Bentazon-8-OH 1	-100.0	-10.0	-26.0	-17.0	254.9	191.0	3.60

Bentazon-8-OH 2	-100.0	-10.0	-34.0	-17.0	254.9	148.0	3.60
