# Supplementary material for: Pesticide distribution and depletion kinetic determination in honey and beeswax: Model for pesticide occurrence and distribution in beehive products
Source: PLoS One. 2019 Feb 20;14(2):e0212631. doi: 10.1371/journal.pone.0212631 (PMC6382162; doi:10.1371/journal.pone.0212631)
Supplement: S2 Table — (PDF) [file pone.0212631.s002.pdf]

S2 Table. MRM transition and optimized parameters for the pesticides analyzed by GC-MS/MS with positive EI ionization.

ID	Precursor Ion (g/mol)	Product Ion (g/mol)	Dwell time (msec)	Collision Energy (v)	*Retention time (min)*
Aldrin	263	193	60	30	*18.42*
	263	191	60	30	
	217	173.1	10	15	
Azaconazole	217	145	10	35	*24.53*
	217	109.1	10	35	
	292.1	264	10	10	
Benfluralin	292.1	160.1	10	20	*11.66*
	292.1	105.2	10	15	
	154	102	15	30	
Biphenyl	153.9	153.2	15	20	*7.08*
	153.9	152.2	15	25	
Bromocyclen	358.7	242.2	10	20	*15.11*
	358.7	143.1	10	20	
Bromocyclen	358.7	242.2	10	20	*15.11*
	358.7	143.1	10	20	
Bromophos-ethyl	358.9	284.8	10	35	*22.47*
Bromophos-ethyl	358.7	331	10	4	
Bromophos-ethyl	358.7	303	10	14	
Bromophos-methyl	330.9	315.9	10	20	
Bromophos-methyl	330.9	285.9	10	34	*20.00*
Bromophos-methyl	329	314	10	12	
Bromopropylate	341	185	10	20	
Bromopropylate	341	183	10	20	*28.64*
Bromopropylate	183	155	10	15	
Bromopropylate	183	76	10	35	
Captafol	151	80	10	10	
Captafol	151	79	10	15	*28.02*
Captafol	79	77	10	10	
Captan	151	80	10	10	
Captan	151	79	10	15	*21.18*
Captan	79	77	10	10	
Chinomethionat	234	206	10	10	*21.86*
Chinomethionat	206	148	10	15	
Chinomethionat	234	206	10	10	*21.86*
Chinomethionat	206	148	10	15	
Chlorbenside	267.9	125	10	8	
Chlorbenside	124.9	99	10	20	*21.78*
Chlorbenside	124.9	89.1	10	20	
Chlorbenside	267.9	125	10	8	
Chlorbenside	124.9	99	10	20	*21.78*
Chlorbenside	124.9	89.1	10	20	
Chlordane, cis-	372.7	266.1	10	25	*22.76*
Chlordane, cis-	272	237	10	20	
Chlordane, trans-	372.7	266.1	10	25	*21.96*
Chlordane, trans-	372.7	264.1	10	25	
Chlorfenapyr	408	59	10	10	*25.27*
Chlorfenapyr	247	227	10	15	

Chlorfenapyr	247	197	10	20	
Chlorfenprop-methyl	195.9	164.8	15	10	*9.94*
Chlorfenprop-methyl	195.9	124.9	15	30	
Chlorfenson	301.8	111.1	10	22	*23.29*
Chlorfenson	174.8	111.1	10	10	
Chlorflurenol-methyl	274	215	10	5	
Chlorflurenol-methyl	215	187	10	12	*22.04*
Chlorflurenol-methyl	215	152	10	24	
Chlorflurenol-methyl	274	215	10	5	
Chlorflurenol-methyl	215	187	10	12	*22.04*
Chlorflurenol-methyl	215	152	10	24	
Chlormefos	233.8	121.1	15	5	
Chlormefos	154	93.1	15	10	*7.71*
Chlormefos	154	65	15	15	
Chlorobenzilate	253	141	10	10	
Chlorobenzilate	139	111	10	15	*25.40*
Chlorobenzilate	139	75	10	30	
Chlorobenzilate	251	139	10	12	
Chloroneb	191	141	20	10	*8.64*
Chloroneb	191	113	20	15	
Chlorpropham	213	171	10	5	*11.03*
Chlorpropham	213	127	10	5	
Chlorpropylat	250.9	139.1	10	15	*25.40*
Chlorpropylat	250.9	111.1	10	35	
Chlorpyrifos-methyl	286	270.9	10	20	*16.53*
Chlorpyrifos-methyl	286	93	10	20	
Chlorthal-dimethyl	301	223	10	25	*19.38*
Chlorthal-dimethyl	299	221	10	25	
Chlorthalonil	265.9	231	10	15	
Chlorthalonil	265.9	133	10	40	*14.79*
Chlorthalonil	263.8	168	10	25	
Chlorthalonil	265.9	231	10	15	
Chlorthalonil	265.9	133	10	40	*14.79*
Chlorthalonil	263.8	168	10	25	
Chlorthion	297	125.1	10	10	*19.69*
Chlorthion	297	109	10	10	
Chlozolinate	330.8	259.1	10	5	*21.31*
Chlozolinate	188.1	147.1	10	15	
Cycloate	154	83.2	15	5	*10.69*
Cycloate	154	71.9	15	5	
DDD, o,p'-	237	165	10	20	
DDD, o,p'-	235	199.1	10	15	*24.33*
DDD, o,p'-	235	165	10	20	
DDD, p,p'-	237	165	10	25	
DDD, p,p'-	235	199.1	10	20	*25.69*
DDD, p,p'-	235	165	10	20	
DDE, o,p'-	248	176	10	30	
DDE, o,p'-	246	211	10	20	*22.43*
DDE, o,p'-	246	176	10	30	
DDE, p,p'-	248	176	10	30	
DDE, p,p'-	246	176	10	30	*24.00*
DDE, p,p'-	246	175.1	10	20	
DDT, o,p'-	237	165	10	20	*25.57*
DDT, o,p'-	235	199.1	10	20	

DDT, o,p'-	235	165	10	20	
DDT, p,p'-	237	165	10	20	
DDT, p,p'-	235	199	10	20	*26.99*
DDT, p,p'-	235	165	10	20	
Desmetryn	212.9	198.3	10	10	
Desmetryn	212.9	141.2	10	15	*15.96*
Desmetryn	212.9	58.2	10	10	
Di-allate I + II	234	192	10	10	*11.87*
Di-allate I + II	234	150	10	20	
Diazinon	304	179	10	15	
Diazinon	179.1	137.2	10	20	*14.39*
Diazinon	179.1	121.1	10	40	
Diazinon	304	179	10	15	
Diazinon	179.1	137.2	10	20	*14.39*
Diazinon	179.1	121.1	10	40	
Dichlobenil	171	136	15	15	*6.73*
Dichlobenil	171	100	15	30	
Dicloran	205.9	175.9	10	5	
Dicloran	205.9	123.9	10	25	*12.56*
Dicloran	175.9	148	10	5	
Dicloran	175.9	140	10	10	
Dicloran	205.9	175.9	10	5	
Dicloran	205.9	123.9	10	25	*12.56*
Dicloran	175.9	148	10	5	
Dicloran	175.9	140	10	10	
Dicofol, o,p-	251	139	15	15	
Dicofol, o,p-	139	111	15	18	*17.57*
Dicofol, o,p-	139	75	15	30	
Dicofol, p,p-	251	139	10	15	
Dicofol, p,p-	139	111	10	18	*19.16*
Dicofol, p,p-	139	75	10	30	
Dieldrin	262.8	228	10	30	
Dieldrin	262.8	193	10	30	*23.80*
Dieldrin	262.8	191	10	30	
Dinobuton	211	117.1	10	15	*20.75*
Dinobuton	211	89.1	10	30	
Diphenylamine	169	168	15	15	
Diphenylamine	169	167	15	20	*10.48*
Diphenylamine	169	141	15	20	
Endosulfan (alpha isomer)	241	206	10	15	
Endosulfan (alpha isomer)	229	194	10	10	*22.55*
Endosulfan (alpha isomer)	195	159	10	5	
Endosulfan (beta isomer)	238.8	204	10	15	
Endosulfan (beta isomer)	229	194	10	10	*25.15*
Endosulfan (beta isomer)	195	159	10	10	
Endosulfan sulfate	387	253	10	5	
Endosulfan sulfate	271.9	237	10	20	*26.76*
Endosulfan sulfate	271.9	117	10	40	
Endrin	281	245	10	20	
Endrin	263	193	10	35	*24.70*
Endrin	263	191	10	35	
Etaconazole	244.9	191.1	10	5	*25.75*
Etaconazole	244.9	172.9	10	15	

Etaconazole	172.8	145	10	15	
Etaconazole	172.8	109.2	10	30	
Etridiazole	210.9	182.9	15	5	
Etridiazole	183	108	15	40	*7.92*
Etridiazole	182.9	139.9	15	15	
Fenchlorphos	285	93	15	25	
Fenchlorphos	284.9	269.9	15	15	*17.27*
Fenchlorphos	284.9	239.9	15	35	
Fenitrothion	277	260	15	5	
Fenitrothion	276.8	260	15	5	
Fenitrothion	276.8	125	15	15	
Fenitrothion	277	260	60	5	
Fenitrothion	276.8	260	60	5	
Fenitrothion	276.8	125	60	15	
Fenson	268	141	10	5	*19.60*
Fenson	141	77.1	10	5	
Fenvalerat (RR + SS)	225	119	70	15	
Fenvalerat (RR + SS)	167	125	70	10	*34.79*
Fenvalerat (RR + SS)	167	89	70	40	
Fenvalerat (RS + SR)	225	119	70	15	
Fenvalerat (RS + SR)	167	125	70	10	*34.37*
Fenvalerat (RS + SR)	167	89	70	40	
Fluchloralin	325.9	63	10	15	
Fluchloralin	306.1	206	10	15	*14.59*
Fluchloralin	306	264	10	5	
Flumethralin	360	314	10	16	
Flumethralin	143	117	10	20	*23.25*
Flumethralin	143	107	10	24	
Fluotrimazole	379	276	10	10	
Fluotrimazole	311	242	10	15	*28.04*
Fluotrimazole	311	165	10	20	
Folpet	260	130	10	15	
Folpet	147	103.1	10	5	*24.56*
Folpet	147	76	10	25	
Fonofos	246.1	137	10	5	*13.81*
Fonofos	246	109.1	10	15	
Genite	304	141.1	10	5	
Genite	302	141	10	5	*22.94*
Genite	141	77	10	5	
HCH alpha isomer	219	183	10	10	
HCH alpha isomer	219	145	10	20	*12.03*
HCH alpha isomer	181	145	10	15	
HCH beta isomer	219	183.1	10	10	
HCH beta isomer	219	145	10	20	*13.25*
HCH beta isomer	181	145.1	10	15	
HCH delta	219	183	10	10	
HCH delta	219	145	10	20	*14.58*
HCH delta	181	145	10	15	
HCH epsilon isomer	219	183	10	10	
HCH epsilon isomer	219	145	10	20	*15.45*
HCH epsilon isomer	181	145	10	15	
Heptachlor	274	239	10	20	
Heptachlor	271.9	236.8	10	25	*16.71*
Heptachlor	271.9	116.9	10	40	

Heptachlor epoxide, cis-	353	282	10	15	*20.64*
Heptachlor epoxide, cis	353	263	10	15	
Heptachlor epoxide, trans-	217	182	10	15	
Heptachlor epoxide, trans-	183	155	10	20	*20.88*
Heptachlor epoxide, trans-	183	119	10	30	
Hexachlorobenzene	283.9	248.8	10	25	*12.31*
Hexachlorobenzene	283.9	213.9	10	35	
Isocarbophos	230	212	10	8	*19.56*
Isocarbophos	136	108	10	14	
Isodrin	262.8	193	10	37	
Isodrin	192.9	157.1	10	25	*19.94*
Isodrin	192.9	123	10	35	
Isofenphos-oxon	228.9	201	10	8	*19.67*
Isofenphos-oxon	228.9	121	10	30	
Isopropalin	280	238	10	10	*20.62*
Isopropalin	280	118.2	10	30	
Jodfenphos	376.9	361.9	10	20	*23.53*
Jodfenphos	376.9	93	10	35	
Leptophos	171	124	10	10	*29.75*
Leptophos	171	77	10	25	
Lindane	218.8	183	10	5	
Lindane	181	109	10	30	*13.39*
Lindane	180.9	145	10	12	
Methoxychlor	227	169	10	25	*28.89*
Methoxychlor	227	141	10	32	
Methyl parathion	263	109.1	10	15	*16.54*
Methyl parathion	263	79.1	10	30	
Mirex	272	237	10	15	
Mirex	272	235	10	25	*29.84*
Mirex	272	117	10	40	
Naled	185	109	15	15	
Naled	145	109	15	25	*5.81*
Naled	109	79	15	5	
Nitrofen	282.9	253	10	10	
Nitrofen	282.9	202.1	10	10	*24.86*
Nitrofen	282.9	162	10	25	
Nitrothal-isopropyl	235.9	194.1	10	5	*19.80*
Nitrothal-isopropyl	235.9	148.1	10	20	
Oxychlordane	387	263	10	14	
Oxychlordane	187	151	10	4	*20.69*
Oxychlordane	185	149	10	4	
Paclobutrazol	236	167	10	20	*22.52*
Paclobutrazol	236	125	10	20	
Pentachloroaniline	265	194	10	20	*15.50*
Pentachloroaniline	265	158	10	35	
Pentachloroanisole	264.8	143	10	35	*12.52*
Pentachloroanisole	264.8	117.1	10	35	
Pentachlorobenzene	250	213	20	15	
Pentachlorobenzene	249.9	214.9	20	20	*8.90*
Pentachlorobenzene	249.9	142	20	40	
Perthane	222.8	179.2	10	20	*25.12*
Perthane	222.8	165.3	10	25	
Phenkapton	375.7	153	10	5	*28.90*

Phenkapton	191	155	10	20	
Phenylphenol, o-	170	141.2	20	20	
Phenylphenol, o-	170	115.1	20	35	*8.79*
Phenylphenol, o-	169	115	20	35	
Phosphamidon II	264	127	10	15	*16.18*
Phosphamidon II	127	109	10	10	
Phthalimid	147	104	15	20	
Phthalimid	147	103	15	20	
Procymidone	283	255	10	10	
Procymidone	283	96	10	10	*21.91*
Procymidone	283	67.1	10	40	
Procymidone	283	255	10	10	
Procymidone	283	96	10	10	*21.91*
Procymidone	283	67.1	10	40	
Profluralin	318.1	237.9	10	15	*13.97*
Profluralin	318.1	199.1	10	10	
Propachlor	176	92	15	20	
Propachlor	120.1	92.1	15	5	*10.30*
Propachlor	120.1	77.1	15	20	
Propanil	217	161	10	8	
Propanil	161	126	10	15	*16.15*
Propanil	161	99	10	25	
Propanil	161	90.1	10	20	
Propanil	217	161	15	8	
Propanil	161	126	15	15	*16.15*
Propanil	161	99	15	25	
Propanil	161	90.1	15	20	
Quintozene	237	143	10	30	
Quintozene	237	119	10	30	*13.61*
Quintozene	295	237	10	20	
Spiromesifen	370	272	10	5	
Spiromesifen	273	255	10	10	*28.38*
Spiromesifen	272	254	10	5	
S421	130	95	15	20	*17.28*
S421	130	60.1	15	35	
TDCPP	381	159	10	12	
TDCPP	381	123	10	24	*26.78*
TDCPP	321	99	10	10	
Tecnazene	215	179	15	15	
Tecnazene	203	83.1	15	25	*10.20*
Tecnazene	202.9	143	15	20	
Tefluthrin, cis-	177	137	10	15	*14.99*
Tefluthrin, cis-	177	127	10	15	
Terbacil	161	144	10	10	*14.68*
Terbacil	161	88.1	10	20	
Terbacil	161	144	10	10	*14.68*
Terbacil	161	88.1	10	20	
Tetradifon	355.7	159	10	10	*29.41*
Tetradifon	353.7	159	10	10	
cis-1,2,3,6-Tetrahydrophthalimid	151	80	20	10	*8.27*
cis-1,2,3,6-Tetrahydrophthalimid	151	79	20	15	
Tetrasul	323.8	253.8	10	25	*26.06*
Tetrasul	251.8	217.2	10	25	

Tetrasul	251.8	182.2	10	35	
Triadimenol	168	70	10	10	*22.04*
Triadimenol	128	65	10	20	
Triadimenol	168	70	10	10	*22.04*
Triadimenol	128	65	10	20	
Tri-allate	269.9	186	10	20	*14.88*
Tri-allate	267.9	184	10	20	
Trichloronate	296.8	269.2	10	10	*19.76*
Trichloronate	296.8	223	10	35	
Trifluralin	306	264	10	10	*11.58*
Trifluralin	263.9	160.1	10	15	
Triphenyl phosphate	326.1	233	10	10	*27.72*
Triphenyl phosphate	326	169	10	30	
Triphenyl phosphate	326	77	10	30	
Vinclozolin	284.8	213	10	10	*16.57*
Vinclozolin	212	172	10	15	
Vinclozolin	212	145	10	20	
Zoxamide	258	187	10	15	*28.07*
Zoxamide	258	159	10	30	
Zoxamide	258	187	10	15	*28.07*
Zoxamide	258	159	10	30	
