# Supplementary material for: Pesticide distribution and depletion kinetic determination in honey and beeswax: Model for pesticide occurrence and distribution in beehive products
Source: PLoS One. 2019 Feb 20;14(2):e0212631. doi: 10.1371/journal.pone.0212631 (PMC6382162; doi:10.1371/journal.pone.0212631)
Supplement: S3 Table — (PDF) [file pone.0212631.s003.pdf]

S3 Table. Linear regression F-test of LogD vs time[a]

	Slope Coefficients	R Square	Critical *F value*	P-value
Acetamipride	0.0003	0.37	1.76	0.28
Clothianidine	0.0003	0.28	0.79	0.47
Imidaclopride	0.0003	0.01	0.03	0.88
Thiaclopride	0.0002	0.15	0.54	0.52
Thiamethoxam	-0.0017	0.13	0.47	0.54
Alachlor	0.0002	0.04	0.14	0.74
Atrazine	0.0005	0.11	0.37	0.59
Metolachlor	0.0005	0.22	0.85	0.42
Chlorantraniliprole	0.0006	0.09	0.30	0.62
chlorpyrifos	0.00002	0.002	0.003	0.96
Fenbutatin oxide	-0.0001	0.02	0.06	0.83
coumaphos	-0.0001	0.21	0.53	0.54
Boscalid	0.00002	0.003	0.01	0.93
Carbendazim	-0.00001	0.001	0.001	0.98
Metoconazole	-0.0001	0.014	0.04	0.85

[a]The null hypothesis states, that the linear slope of LogD vs. time equals zero. The F-test enables to determine, whether the slope is significantly different form zero. For calculated p value > 0.05, the linear slope was statistically defined as zero.

[b]Linear slope

[c]R^2, coefficient of determination, defined as the proportion of the variance in the dependent variable (LogD) that is predictable from the independent variable (time).

[d]F statistics, test statistic for testing the statistical significance of the model.

[e]P-value; when the calculated p value is above the significance level of 0.05, then the null hypothesis is accepted as true, hence, the linear slope equals to zero.
